# Reticulocalbin-2 (RCN2) as a Context-Dependent CREC-Family Modulator: Gene Expression and Regulation, Molecular Biology, Disease Relevance, and Translational Challenges

**DOI:** 10.3390/genes17101226

**Published:** 2026-10-02

**Authors:** Abdullah A. A. Alghamdi

**Affiliations:** Department of Biology, Faculty of Science, Al-Baha University, Al-Baha 65779, Saudi Arabia; aaa.alghamdi@bu.edu.sa

**Keywords:** RCN2, endoplasmic reticulum, calcium homeostasis, signal transduction, protein interactions, cancer, Raptin

## Abstract

Reticulocalbin-2 (RCN2), encoded by the human *RCN2* gene (HGNC:9935) and historically identified as E6-binding protein, is a CREC-family EF-hand Ca^2+^-binding protein. Although first characterized as an endoplasmic reticulum (ER)-associated protein, current evidence extends RCN2 biology to splice-variant and localization complexity, protein interactions, regulatory and signaling networks, extracellular activity, cancer, non-malignant disease, and the RCN2-derived hormone Raptin. This review synthesizes the fragmented and methodologically uneven RCN2 literature within an evidence-stratified framework. Mechanistic evidence, including direct biochemical assays and RCN2-directed functional studies, supports selected interaction and signaling relationships, whereas omics, database-derived and single-context observations remain hypothesis-generating. Across disease settings, RCN2 is best interpreted as a context-dependent modulator rather than a uniform oncogenic driver, validated biomarker or established therapeutic target. Translational progress will require molecular-form-specific assays, disease-specific validation and clinically grounded models.

## 1. Introduction

Calcium-dependent signaling in the endoplasmic reticulum (ER) and secretory pathway provides a framework for linking luminal Ca^2+^ storage and buffering with protein folding and maturation, organellar communication, and adaptive stress responses [1,2,3]. Many ER and secretory-pathway Ca^2+^-binding proteins are therefore not merely passive luminal buffers, because calcium-dependent folding, quality control, and cargo maturation and trafficking processes help maintain proteostasis across changing physiological and pathological conditions [2,4,5]. This broader view is especially relevant because ER stress activates the unfolded protein response (UPR), a homeostatic and adaptive signaling network that links protein-folding burden and ER Ca^2+^ homeostasis to cell-fate decisions, metabolic and inflammatory programs, and tissue-context-dependent remodeling [6,7]. ER contacts with mitochondria and other organelles further extend this framework; in particular, ER–mitochondria interfaces link ER Ca^2+^ release and handling to mitochondrial participation in local and global intracellular Ca^2+^ signaling, bioenergetic adaptation, and stress-responsive survival or cell-death outcomes [8,9,10].

Within this biological landscape, reticulocalbin-2 (RCN2) belongs to the CREC family of multiple EF-hand, low-affinity Ca^2+^-binding proteins associated with the mammalian secretory pathway [11,12]. This family-level placement is important because selected CREC members have already been examined in defined secretory-pathway or disease-associated contexts; for example, Cab45, encoded by *SDF4*, mediates Ca^2+^-dependent trans-Golgi network cargo sorting, whereas calumenin, encoded by *CALU*, has broader calcification- and cancer-related relevance [13,14]. By contrast, RCN2 has received less integrated critical synthesis as an individual molecule, even though its canonical ER-associated identity was established early and later CREC-family synthesis summarized its aliases, variant-related complexity, interaction partners, and disease-associated observations [12,15]. To our knowledge, no review dedicated specifically to RCN2 has been identified. This imbalance should not be interpreted as evidence that RCN2 is biologically less relevant than other CREC-family members; rather, it reflects how the RCN2 literature has developed through distinct historical entry points, including papillomavirus E6-binding biology, CREC-family classification, and later ERC-55 interaction analyses [11,16,17].

The need for a focused RCN2 review has become stronger because the literature now extends beyond RCN2′s original ER-associated identity while remaining uneven in mechanistic depth [15,17]. Subsequent work has broadened the RCN2 literature to include reported protein interactions, regulatory and signaling associations, calcium- and stress-associated signaling, extracellular activity, cancer biology, and non-malignant disease contexts, with the discovery of Raptin adding a distinct RCN2-derived secreted-hormone axis [18,19,20,21,22]. However, the available studies differ substantially in evidential strength, ranging from direct biochemical evidence and RCN2-directed perturbation or rescue studies to candidate observations emerging from broader transcriptomic, proteomic, or secretomic discovery analyses [17,19,20,23,24,25,26]. This heterogeneity makes it necessary to distinguish experimentally supported RCN2 biology from candidate associations and to avoid reducing RCN2 either to a simple ER-resident Ca^2+^-binding protein or to an overgeneralized disease marker.

This review maps the evidence supporting RCN2 molecular forms, interactions, regulatory mechanisms, context-specific signaling, disease associations, and translational limitations.

## 2. Literature Search and Evidence Appraisal

This article was developed as a structured narrative review. Relevant literature was identified through searches of Web of Science, PubMed, and Google Scholar using the following terms: “RCN2,” “reticulocalbin-2,” “reticulocalbin 2,” “ERC-55,” “E6-binding protein,” “E6BP,” “VAF1,” “TCBP49,” “taipoxin-associated calcium-binding protein 49,” and “Raptin.” Targeted searches additionally combined the principal RCN2 terms with concepts related to protein interactions, calcium and endoplasmic reticulum biology, cellular stress, cancer, vascular disease, calcification, inflammation, bone and cartilage biology, metabolism, and neurological or translational relevance. Database-specific search syntax was adapted to the functionality of each platform. Structured core and targeted Boolean searches were applied in PubMed and Web of Science Core Collection, whereas Google Scholar was used as a supplementary broad-discovery source using the core alias-based query because extended targeted Boolean combinations yielded predominantly low-specificity results. No lower publication-date limit was applied, and the literature search was last updated on 23 September 2026. No formal language restriction was applied at the search stage; however, the evidentiary synthesis was limited to publications that could be evaluated reliably in English. The study by Zhan et al. (2008) [27], although not indexed in PubMed, was retained because an English-language full-text version was available and could be appraised directly. Reference lists of relevant primary studies and reviews were screened, and forward citation searches were conducted in Google Scholar to identify additional potentially eligible publications. The core PubMed and Web of Science searches retrieved 245 records, of which 135 remained after removal of 110 cross-database duplicates. Screening of the first 200 relevance-ranked Google Scholar results yielded nine additional unique records, resulting in 144 unique records for evidence appraisal; 70 were retained as RCN2-specific evidence, 21 as contextual or background evidence, and 53 were excluded. The exact database-specific search strings, search dates, and retrieval and retention counts are provided in Table S1.

A single reviewer identified potentially relevant studies, assessed eligibility, and appraised the evidence. Primary peer-reviewed studies were prioritized when they directly examined RCN2, its historical aliases or experimentally characterized mammalian homologues, RCN2 splice variants, extracellular or exosome-associated RCN2, or the RCN2-derived hormone Raptin. Selected reviews and non-RCN2 studies were used to establish historical, family-level, mechanistic, or disease-context background and were not treated as RCN2-specific evidence.

Studies were excluded from the RCN2 evidentiary synthesis if they concerned other CREC-family members without RCN2-specific evidence or used the RCN2 designation for an unrelated gene or protein in a non-mammalian organism. Claims that could not be traced to an identifiable primary source were not incorporated into the RCN2-specific evidentiary synthesis. Non-peer-reviewed records were not used as primary evidence in the narrative synthesis. Articles identified as retracted or subject to an expression of concern affecting the relevant RCN2 evidence were excluded. Publication status was checked during the final evidence appraisal using PubMed and publisher records. No retracted RCN2-specific studies were identified (*n* = 0); one RCN2-specific article subject to an expression of concern was identified and excluded from the evidentiary synthesis (*n* = 1). Transcriptomic, proteomic, secretomic, computational, database-derived, and other high-throughput observations, as well as unvalidated genetic associations, were retained when relevant, but their interpretation was calibrated to the level of RCN2-specific validation. Unvalidated observations were treated as contextual or hypothesis-generating, whereas claims of biological function or causality required RCN2-directed functional evidence.

Evidence was classified as foundational molecular/genetic, direct biochemical, cellular physical association or multiprotein-complex membership, functional, clinical observational, or discovery-level. The functional category was assigned to studies that used RCN2-directed perturbation, rescue, pathway inhibition, or genetic models to test biological consequences. Combined evidence categories were assigned only when more than one evidence type was independently supported. In grouped table rows, the combined designation summarizes the collective evidence and does not imply that every listed study demonstrated each evidence type. Interpretation also considered the experimental method, species, biological system, cell or tissue type, RCN2 molecular form and localization, causal support, and the availability of independent replication.

BioGRID was additionally consulted to identify curated candidate protein associations involving RCN2. The Raw Export worksheet preserves the original record-level data from the *RCN2* gene-specific export of BioGRID release 5.0.260, accessed on 19 August 2026, without filtering, deduplication, or reclassification. The dataset is provided as File S1. A separately generated partner-level summary is provided as File S2. BioGRID-derived partners were treated as candidate associations rather than as evidence of confirmed direct RCN2 binding.

## 3. History and Nomenclature of RCN2

### 3.1. Early Identification, Historical Names, and Mammalian Homologues

RCN2 first entered the literature through protein-centered studies rather than gene-based annotation, and one of the earliest defining reports identified ERC-55 as a previously unrecognized ER-localized calcium-binding protein [15]. A second naming route emerged soon afterward when the same protein was identified in papillomavirus research as E6-binding protein (E6BP), and Chen and colleagues explicitly stated that E6BP was identical to the putative calcium-binding ER protein ERC-55 [16]. The historical nomenclature broadened further in 1997, when Imai and colleagues described VAF1 as a mouse homologue of human ERC-55 and reported that it specifically interacted with the vitamin D receptor (VDR) among the nuclear receptors tested [28]. In parallel, the rat neurotoxin-related literature used the designation taipoxin-associated calcium-binding protein 49 (TCBP49) for a related reticular calcium-binding protein identified in the taipoxin and neuronal-pentraxin literature [29]. Sequence-level support for this relationship is provided by the rat TCBP49 sequence reported by Dodds et al. [30], associated with GenBank nucleotide accession U15734. The corresponding protein record (AAA80197.1) is currently linked by NCBI to the rat *Rcn2* gene, supporting the identification of TCBP49 as the rat RCN2 counterpart [31]. These early studies show that the present-day RCN2 literature developed through several partially overlapping naming traditions, encompassing the human designations ERC-55 and E6BP together with the species-linked mammalian designations VAF1 and TCBP49. This nomenclatural fragmentation may complicate interpretation of the early literature unless species and terminology are tracked explicitly [15,16,28,29].

### 3.2. RCN2 Within the CREC Family

The broader conceptual placement of this protein became clearer when Honoré and Vorum proposed the CREC family as a group of multiple EF-hand, low-affinity Ca^2+^-binding proteins localized to the secretory pathway of mammalian cells [11]. That early family-level framework placed ERC-55/E6BP and TCBP49 within the broader CREC-family context, but did not itself establish that TCBP49 corresponds to rat *Rcn2*. The sequence-level mapping described in Section 3.1 provides the basis for linking TCBP49 specifically to rat *Rcn2*, while the CREC-family framework remains useful for interpreting the historically distinct ER calcium-binding, papillomavirus E6-binding, and taipoxin-associated studies [11,29]. A later review refined this classification further by organizing the CREC proteins around five genes—*RCN1*, *RCN2*, *RCN3*, *SDF4*, and *CALU*—and explicitly assigning ERC-55 to the *RCN2* gene product [12]. Terminological care is required because some CREC-family studies use reticulocalbin to denote RCN1 rather than RCN2/ERC-55. For example, Hansen and colleagues identified fibulin-1C, C1 esterase inhibitor, and GRP75 as partners of calumenin and reticulocalbin, but this study should not be interpreted as direct RCN2/ERC-55-specific interaction evidence [32]. Accordingly, the CREC-family framework did more than provide background classification by supplying the conceptual bridge needed to interpret the historical names and related mammalian designations linked to RCN2 within a unified CREC-family context [11,12].

### 3.3. Transition from Historical Names to Current RCN2 Nomenclature

The transition from protein-centered aliases to modern gene-based nomenclature became explicit when Wang and colleagues used in situ hybridization to assign the gene for ERC-55 (*RCN2*) to chromosome 15q22.33–q24.1, thereby formally linking the older protein name with the newer gene designation in a single report [33]. This historical cytogenetic assignment has since been refined; in the current GRCh38.p14 RefSeq annotation, human *RCN2* (HGNC:9935; NCBI Gene ID: 5955) is located at chromosome band 15q24.3 (NC_000015.10:76,931,749–76,954,393). Even after that bridge had been established, the older naming systems remained active in the literature, as illustrated by Chen and colleagues in 1998, who continued to use E6BP or ERC-55 while mapping the papillomavirus E6-binding region within the protein [34]. This persistence was also evident in HPV-related functional and translational studies published before and around 2000, including Liu et al. in 1999 and Beerheide et al. in 1999 and 2000, all of which continued to refer to the protein as E6BP, in some cases explicitly noting its equivalence to ERC-55 [35,36,37]. At the same time, the TCBP49 designation persisted in the rat neuronal-pentraxin/taipoxin literature beyond 1997, as illustrated by Kirkpatrick and colleagues in 2000 [38]. These studies indicate that the emergence of RCN2 as the preferred modern name was gradual rather than immediate, and that accurate reading of the early literature still requires explicit recognition of the parallel historical names ERC-55 and E6BP together with the species-linked designation TCBP49 [33,37,38].

## 4. Molecular and Cellular Biology of RCN2

### 4.1. Gene Architecture, Transcript Diversity, and Protein Features

Human *RCN2* (HGNC:9935; NCBI Gene ID: 5955; Ensembl ENSG00000117906) is a protein-coding gene with eight exons in the current NCBI annotation [39]. Two reviewed RefSeq protein-coding transcript variants are currently maintained. Transcript variant 1, NM_002902.3, is the predominant transcript and corresponds to Ensembl ENST00000394885.8; these transcripts constitute the current MANE Select pair [39,40]. NM_002902.3 encodes the 317-amino-acid reticulocalbin-2 isoform a precursor (NP_002893.1). Transcript variant 2, NM_001271837.2 (corresponding to ENST00000320963.10), contains an additional in-frame coding exon relative to variant 1 and encodes the longer isoform b precursor (NP_001258766.1) [39]. These current accession-based annotations should be distinguished from the experimentally described ERC-55 splice-variant nomenclature. Ludvigsen et al. demonstrated several ERC-55 splice variants, including the cytosolic, cytoskeleton-associated ERC-55-C form [17]; however, a one-to-one correspondence between ERC-55-C and either of the currently reviewed RefSeq isoforms has not been established by the available annotation and primary-study evidence.

At the protein level, the foundational ERC-55 study characterized the *RCN2* gene product as an endoplasmic-reticulum Ca^2+^-binding protein with an apparent molecular mass of approximately 55 kDa [15]. The study reported an amino-terminal signal sequence, six EF-hand motifs, and a carboxyl-terminal His-Asp-Glu-Leu (HDEL) sequence, establishing the principal molecular features subsequently associated with canonical RCN2. For canonical human RCN2 (Q14257), the UniProt record evaluated by Morikawa et al. annotated the signal peptide as residues 1–22; however, N-terminomic analysis detected an RCN2 neo-N terminus at residue 26, consistent with a SignalP 6.0 prediction of residues 1–25, indicating that the exact cleavage boundary remains incompletely resolved [41]. It also described ERC-55 as being encoded by a single-copy gene and detected an approximately 1900-base mRNA with broad expression across human tissues [15]. Consistent with this broad rather than lineage-restricted expression pattern, ERC-55 mRNA was detected in pancreas, kidney, skeletal muscle, liver, lung, placenta, brain, and heart, while subsequent protein-level analyses detected a 55-kDa ERC-55 band across the human tissues examined, with particularly strong expression in testis, brain, and lung [15,17]. More recent disease-oriented studies continue to describe RCN2 primarily as an ER-associated or ER-luminal EF-hand Ca^2+^-binding protein [24,42].

Promoter-level regulation of *RCN2* has been demonstrated in several cellular contexts, although the experimentally interrogated promoter regions remain study-specific rather than defining a single universally validated core promoter. In HCC cells, YY1-dependent transcriptional activation was supported by promoter-reporter and chromatin-immunoprecipitation (ChIP) assays [42]. In NPC cells, both YY1 and GSC were shown by promoter-reporter and ChIP analyses to bind predicted *RCN2* promoter regions and contribute to *RCN2* transcription [20]. In human endothelial cells, SP1-dependent promoter activation was supported by reporter assays and electrophoretic mobility-shift analysis at a predicted SP1-binding site reported at positions 1803–1813 within the promoter sequence analyzed in that study; endogenous-locus occupancy was not tested by ChIP [43]. These findings support context-specific promoter regulation and are discussed mechanistically in Section 5.2.

Population-level constraint data provide additional context for the interpretation of human *RCN2* variation. Current gnomAD v4.0 gene-constraint metrics reported for the MANE-associated ENST00000394885 transcript (pLI = 0; LOEUF = 1.26) do not indicate marked constraint against predicted loss-of-function variation [44]. These gene-level metrics, however, cannot establish the functional or clinical significance of individual variants. Accordingly, the uncommon rs144252373/p.L57I variant identified in the Army STARRS single-variant analysis [45] remains an association-level observation requiring independent replication and functional characterization.

At the paralog level, *RCN2* is one of five mammalian CREC-family genes, together with *RCN1*, *RCN3*, *SDF4*, and *CALU* [12], as discussed in Section 3.2. These related CREC-family genes share structural features but should not be assumed to be functionally interchangeable.

The human *RCN2* gene encodes the protein historically characterized as ERC-55 [33]. The original ERC-55 study defined the protein as a human endoplasmic reticulum Ca^2+^-binding protein that migrates with an apparent molecular mass of approximately 55 kDa [15]. That study further reported that ERC-55 is encoded by a single-copy gene and an approximately 1900-base mRNA showing a broadly distributed expression pattern across multiple human tissues. At the sequence level, ERC-55 was shown to contain an amino-terminal signal sequence, six EF-hand motifs, and a carboxyl-terminal His-Asp-Glu-Leu (HDEL) sequence, providing the core molecular features that later studies continued to associate with RCN2 [15]. Subsequent CREC-family synthesis and variant-focused analysis refined this basic identity by indicating that the originally cloned ERC-55 precursor corresponds to a 317-amino-acid RCN2 product, while also recognizing additional transcript complexity [12,17]. The available expression data support broad rather than lineage-restricted expression of RCN2/ERC-55, because ERC-55 mRNA was detected in pancreas, kidney, skeletal muscle, liver, lung, placenta, brain, and heart, and later protein-level analyses detected a 55-kDa ERC-55 band across all examined human tissues with particularly strong expression in testis, brain, and lung [15,17]. More recent disease-oriented studies have continued to introduce RCN2 using this same basic molecular identity, describing it as an ER-associated or ER-luminal Ca^2+^-binding protein with EF-hand-related structural features [24,42]. These studies define RCN2 as a broadly expressed, low-abundance, ER-associated Ca^2+^-binding gene product whose core molecular identity was established in early biochemical and cell-biological work and later refined through gene mapping, CREC-family classification, and splice-variant analysis [15,17,33].

### 4.2. Subcellular Localization and Trafficking

The foundational cell-biological characterization identified ERC-55 as a resident ER protein and localized it predominantly to the rough ER and nuclear envelope, with additional labeling in the intermediate compartment between the ER and Golgi [15]. ER retention was shown to depend in part on the carboxyl-terminal HDEL motif, because deletion of this tetrapeptide caused slow secretion of the mutant protein, whereas wild-type ERC-55 was not detectably secreted under the same pulse-chase conditions [15]. The slow secretion of the HDEL-deleted mutant led the original study to suggest that ERC-55 retention is not explained solely by the terminal retrieval motif and may also involve additional protein-based retention or retardation mechanisms within the ER system [15]. Later CREC-family framing retained this ER-centered view by placing ERC-55/RCN2 among secretory-pathway Ca^2+^-binding proteins, while also recognizing that CREC proteins can differ in localization and trafficking behavior [11,12]. More recent RCN2-focused studies have continued to describe RCN2 as mainly ER-localized or ER-luminal, indicating that the canonical ER-centered identity remains the dominant framework for interpreting the protein [24,43]. This localization profile became more complex with evidence for ERC-55-C, a splice-variant-derived form that showed labeling in the ER, nuclear envelope, and cytosol and was further enriched in the cytoskeletal protein fraction by subcellular fractionation [17]. The available data therefore support a cautious working model in which canonical RCN2/ERC-55 is predominantly ER-associated, whereas variant-dependent or context-dependent forms may extend its distribution beyond the classical ER lumen [15,17]. Consistent with this broader view, a recent study detected RCN2 in bone marrow supernatant and in concentrated cellular supernatant and reported that macrophages secreted RCN2 in response to mechanical loading, suggesting that under specific physiological conditions RCN2 can also display extracellular behavior rather than remaining strictly confined to the ER [19].

### 4.3. Structural Features, Calcium-Binding Properties, and Isoform-Related Considerations

The original ERC-55 study provided direct biochemical support for the Ca^2+^-binding identity of RCN2 by demonstrating ^45^Ca^2+^ binding by recombinant ERC-55 in vitro [15]. CREC-family reviews later described ERC-55/RCN2 as a multiple EF-hand, low-affinity Ca^2+^-binding protein of the secretory pathway, but this family-level framing should be interpreted alongside the direct RCN2/ERC-55 biochemical data rather than as a substitute for them [11,12]. An additional layer of structural and cellular complexity comes from alternative splicing, because later analysis identified several ERC-55 transcript variants and provided direct evidence for ERC-55-C as a cytoskeleton-associated cytosolic variant [17]. This variant evidence indicates that the biology of RCN2 should not be reduced entirely to the originally described ER-resident precursor, although the canonical ER-associated form remains the best-established molecular reference point [15,17]. Contemporary studies in cancer and stress-related contexts continue to refer to RCN2 as an EF-hand Ca^2+^-binding protein mainly associated with the ER, showing that modern functional studies still rely on the core molecular identity established in the foundational literature [20,24,42]. Taken together, the available evidence supports a model in which RCN2 is best understood as a low-abundance, EF-hand-containing, Ca^2+^-binding protein with a predominant ER-associated form and additional splice-variant complexity whose full structural and functional implications remain incompletely resolved [12,15,17].

Although RCN2 was initially characterized as an endoplasmic reticulum-associated Ca^2+^-binding protein, accumulating evidence suggests that its biological significance extends beyond a purely resident ER role, as discussed in the following sections. The canonical protein architecture, major molecular forms, and principal evidence-supported trafficking and subcellular localization states of RCN2 are summarized in Figure 1. Throughout Figure 1, Figure 2 and Figure 3, extracellular RCN2 and Raptin are shown as distinct molecular/signaling categories and are not intended to represent mutually exclusive extracellular states.

## 5. Molecular Interactions and Signaling Networks of RCN2

### 5.1. Experimentally Supported Partner Proteins

For the purposes of this review, reported RCN2 partners are classified as direct biochemical binders, cellular physical associations or members of multiprotein complexes, and preliminary or high-throughput candidate associations. The database-scale candidate interactome is not reproduced in detail here; instead, this subsection focuses on primary-study evidence for which the interaction mode and experimental context can be evaluated directly.

Under the historical designation E6-binding protein/ERC-55, RCN2 was identified as a cellular protein that directly bound selected papillomavirus E6 proteins in yeast two-hybrid and reconstituted glutathione S-transferase (GST)-fusion assays [16]. Subsequent mutational mapping identified an α-helical motif sufficient for association with E6, thereby defining an important structural determinant of the interaction [34]. These findings do not, however, establish direct binding between RCN2 and the cellular ubiquitin ligase E6-associated protein (E6AP), because E6 may bridge the proteins within a larger complex [16]. A later human papillomavirus type 16 (HPV16) E6 mutational analysis further cautioned against assigning a simple functional consequence to E6BP/ERC-55 binding, because E6 mutants showed dissociation between E6BP binding and resistance of primary human keratinocytes to serum- and calcium-induced terminal differentiation [46]. Natural HPV16 E6 variants also showed variable E6BP/ERC-55-binding activity in functional assays relevant to the carcinogenic potential of E6 [47].

Subsequent studies identified direct RCN2 interactions with intracellular signaling regulators in distinct biological settings. In human esophageal squamous cell carcinoma (ESCC) models, GST pull-down assays supported direct binding between RCN2 and protein phosphatase 2A catalytic subunit alpha (PPP2CA), whereas immunoprecipitation, immunofluorescence, and functional rescue experiments established the cellular and functional relevance of this interaction [24]. Within the same mechanism, UBR5 acted as a functional RCN2-associated E3-ligase mediator required for PPP2CA ubiquitination and degradation, although purified binary binding between RCN2 and UBR5 was not established at the same evidential level as the RCN2–PPP2CA interaction [24]. In a developmentally distinct murine context, GST pull-down assays supported direct binding between RCN2 and serine/threonine kinase 40 (STK40), and cellular co-immunoprecipitation demonstrated an STK40-associated RCN2 complex in mouse embryonic stem cells [48]. A later *Stk40*-knockout mouse study identified RCN2 by GST–STK40 affinity purification and provided physiological support for an STK40-associated ERK/MAPK signaling relationship, although *Rcn2* itself was not genetically perturbed in the lung model [49].

Biochemical screening using recombinant ERC-55 identified an additional group of direct in vitro partners. Surface plasmon resonance demonstrated that S100A6 bound recombinant ERC-55 in a strongly Ca^2+^-dependent manner, whereas S100P also bound ERC-55 and required Ca^2+^ under the tested conditions. Lactoferrin and peroxiredoxin 6 likewise bound recombinant ERC-55 in vitro; however, lactoferrin displayed only modest Ca^2+^ dependence, whereas PRDX6 binding was not clearly Ca^2+^-dependent. These findings establish direct biochemical binding under defined in vitro conditions but do not independently demonstrate endogenous cellular interactions or defined physiological roles [17].

A broader group of partners is supported by cellular physical-association evidence rather than purified binary-binding assays. In hepatocellular carcinoma (HCC) cells, RCN2 physically associated with epidermal growth factor receptor (EGFR) and supported epidermal growth factor (EGF)-dependent receptor activation [18]. In nasopharyngeal carcinoma (NPC) cells, endogenous co-immunoprecipitation demonstrated a physical association between RCN2 and calreticulin (CALR) [20]. In pancreatic cancer-associated fibroblasts (CAFs), mass spectrometry and co-immunoprecipitation supported a physical association between microfibril-associated protein 5 (MFAP5) and RCN2 [50]. Collectively, these studies support context-specific cellular associations but do not demonstrate purified direct contact between RCN2 and EGFR, CALR, or MFAP5 [18,20,50].

Cellular-association evidence also extends beyond cancer-related systems. In primary mouse chondrocytes studied in an excessive-mechanical-loading model, RCN2 and signal transducer and activator of transcription 3 (STAT3) co-immunoprecipitated, supporting a cellular physical association; however, purified binary binding was not demonstrated, and the finding should not be generalized as a universal RCN2–STAT3 interaction across tissues or species [51]. RCN2 also co-immunoprecipitated and colocalized with SUN1 and SUN2 at the nuclear envelope in the studied mammalian-cell system, although the functional significance of these associations remained unresolved [52]. In transfected human-cell systems, RCN2 physically associated with phosphatidylinositol glycan anchor biosynthesis class X (PIGX) and participated in a PIGX-centered complex containing RCN1, PIGX, and RCN2. The physical-association and complex-architecture experiments were conducted in 293T cells, whereas T-47D and BT-549 breast-cancer cells provided the related growth and gene-expression evidence; thus, the RCN2–PIGX association was not directly demonstrated in the breast-cancer cell lines examined. Moreover, because *PIGX* depletion markedly reduced the RCN1–RCN2 co-immunoprecipitation signal, the study does not establish a PIGX-independent binary association between RCN1 and RCN2 [53].

Beyond its canonical ER-associated context, extracellular RCN2 was investigated as a secreted signaling factor. In mouse adipocyte models, neuropilin 2 (NRP2) and integrin β1 (ITGβ1) were identified as components of a functional RCN2 receptor complex through RCN2-based membrane-protein capture and liquid chromatography–tandem mass spectrometry in 3T3-L1 cells, with cellular associations involving RCN2, NRP2, and ITGβ1 further supported in transfected HEK293T cells. Loss-of-function experiments supported a functional requirement for both receptor components, although independently purified binary binding of RCN2 to either NRP2 or ITGβ1 was not demonstrated [19]. Raptin represents a further and conceptually distinct interaction class because it is a cleaved and secreted RCN2-derived hormone rather than canonical full-length RCN2 itself. Raptin was reported to bind metabotropic glutamate receptor 3 (GRM3) in neurons of the hypothalamic paraventricular nucleus and the gastric muscular layer; however, these findings do not establish GRM3 as a receptor for canonical full-length, ER-associated RCN2 [21].

Several historical reports broaden the potential RCN2 partner spectrum but remain constrained by species, experimental system, and legacy nomenclature. A mouse calcium-binding protein historically designated VAF1 and described as a homologue of human ERC-55 was reported to interact with VDR in a yeast two-hybrid system and in vitro assays; however, these findings do not by themselves establish a physiological interaction between human RCN2 and VDR [28]. TCBP49 was originally identified and purified from rat brain by affinity chromatography on immobilized-taipoxin columns [30]. Subsequent work placed TCBP49 within a biochemical interaction network involving the neuronal pentraxins [29], with neuronal pentraxin 1 (NP1) and neuronal pentraxin 2 (NP2) subsequently reported to associate biochemically with TCBP49 [38]. Because these findings are restricted to specific species and experimental systems, their relevance to the human RCN2 interaction network remains to be established.

Preliminary evidence links RCN2 to the stromal interaction molecule 1 (STIM1)–calcium release-activated calcium channel protein 1 (ORAI1) complex. Recovery of RCN2 from a tandem-affinity-purified STIM1–ORAI1 complex and its colocalization with STIM1 in the ER support a preliminary complex-level association or proximity rather than direct binary binding [27]. The evidence-stratified intracellular interaction network and extracellular receptor-associated relationships involving RCN2 or RCN2-derived Raptin are summarized in Figure 2.

**Figure 2:**
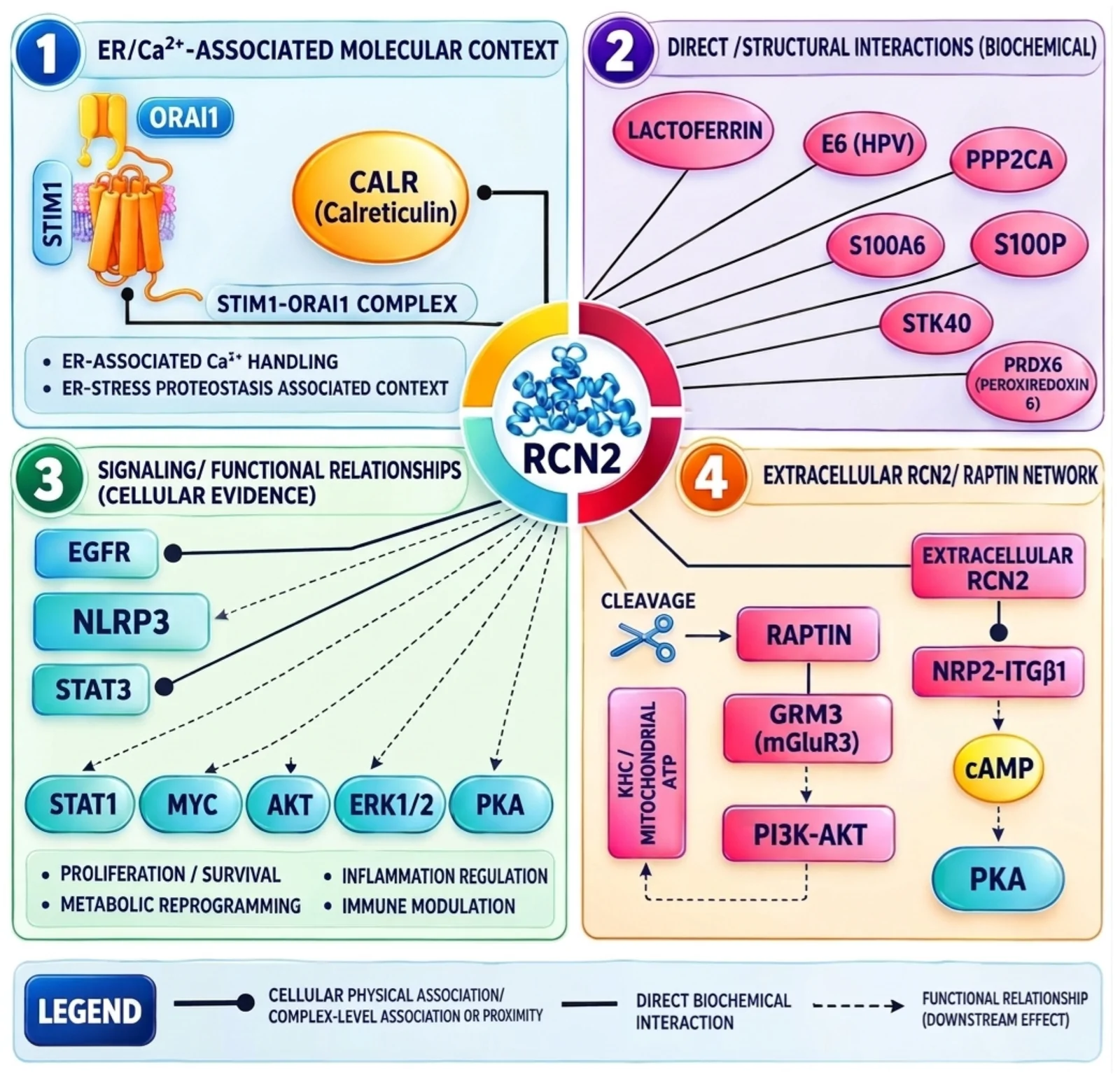
Evidence-stratified intracellular and extracellular RCN2 interaction framework. RCN2-related molecular interactions and signaling relationships are organized into four sections: (1) ER/Ca^2+^-associated molecular context, including the RCN2–CALR association and preliminary association/proximity with the STIM1–ORAI1 complex; (2) direct biochemical interactions supported by binding assays; (3) cellular physical associations and context-dependent downstream functional relationships; and (4) extracellular RCN2 and RCN2-derived Raptin signaling. Extracellular RCN2 acts through an NRP2–ITGβ1 receptor complex with downstream cAMP–PKA signaling, whereas Raptin binds GRM3 and signals through PI3K–AKT to KHC-dependent mitochondrial ATP production. The route responsible for extracellular RCN2 release remains unresolved. Edge styles denote direct biochemical interaction, cellular physical association/complex-level association or proximity, or functional downstream relationship. Downstream relationships in Section 3 represent distinct experimental contexts and should not be interpreted as a single linear signaling pathway. Abbreviations: AKT, protein kinase B; ATP, adenosine triphosphate; CALR, calreticulin; cAMP, cyclic adenosine monophosphate; EGFR, epidermal growth factor receptor; ER, endoplasmic reticulum; GRM3, metabotropic glutamate receptor 3; HPV, human papillomavirus; ITGβ1, integrin β1; KHC, kinesin heavy chain; NLRP3, NLR family pyrin domain containing 3; NRP2, neuropilin 2; ORAI1, calcium release-activated calcium channel protein 1; PI3K, phosphoinositide 3-kinase; PKA, protein kinase A; PPP2CA, protein phosphatase 2A catalytic subunit alpha; PRDX6, peroxiredoxin 6; RCN2, reticulocalbin-2; STAT1, signal transducer and activator of transcription 1; STAT3, signal transducer and activator of transcription 3; STIM1, stromal interaction molecule 1; STK40, serine/threonine kinase 40.

BioGRID is a curated repository of experimentally reported protein, genetic, and chemical interactions derived from both focused and high-throughput studies [54,55]. To complement the individually appraised interactions discussed above, the *RCN2* gene-specific export from BioGRID release 5.0.260, accessed on 19 August 2026, was examined. The dataset comprised 365 interaction records representing 327 unique associated partner entries across organisms, including 303 unique human candidate partners. The complete record-level export is provided as File S1, whereas the partner-level summary, including the predefined review-specific prioritization criteria, is provided as File S2. Because BioGRID records encompass heterogeneous experimental associations, including direct binding, multiprotein-complex membership, experimental proximity, and other context-dependent relationships, database inclusion or prioritization was not considered evidence of direct RCN2 binding without supporting study-level evidence.

### 5.2. Upstream Regulators of RCN2 Expression

Available evidence indicates that *RCN2* expression and protein abundance are regulated at multiple levels, including transcriptional control, post-transcriptional regulation, and post-translational protein stabilization, and are further influenced by inflammatory, mechanical, and metabolic stimuli. These regulatory modes should be distinguished carefully because direct promoter regulation, miRNA-mediated targeting, altered protein turnover, and stimulus-associated changes in expression represent mechanistically distinct forms of upstream control.

Among the transcriptional regulators currently linked to *RCN2*, Yin Yang 1 (YY1), goosecoid homeobox (GSC), and specificity protein 1 (SP1) are supported by direct promoter-level experimental evidence. In HCC cells, YY1 directly promoted *RCN2* transcription, as supported by wild-type and binding-site-mutant promoter-reporter assays, chromatin immunoprecipitation, and siRNA-mediated *YY1* knockdown, which reduced *RCN2* mRNA and protein expression [42]. Importantly, YY1-dependent promoter regulation was independently supported in NPC cells, where knockdown of either *YY1* or *GSC* reduced *RCN2* expression, while dual-luciferase reporter assays supported promoter-level regulation and chromatin-immunoprecipitation assays demonstrated binding of both factors to the predicted *RCN2* promoter region [20]. These findings support YY1 as a promoter-level regulator of *RCN2* in two distinct tumor contexts, whereas GSC promoter regulation is supported in NPC. *RCN2*-overexpression rescue experiments in NPC further supported RCN2 as a functional downstream mediator of YY1/GSC perturbation [20]. In human umbilical-vein endothelial cells, forced SP1 expression increased *RCN2* promoter-driven reporter activity, while electrophoretic mobility-shift assays using *RCN2* promoter-derived probes supported SP1 binding to active promoter regions [43]. However, because the SP1 study did not examine SP1 occupancy at the endogenous *RCN2* locus by chromatin immunoprecipitation, its evidence is methodologically distinct from the in-cell chromatin-occupancy evidence reported for YY1 and GSC [20,42,43]. Collectively, these findings support context-specific transcriptional activation of *RCN2* by YY1, GSC, and SP1 but do not establish that any of these transcription factors universally regulates *RCN2* across tissues or cellular contexts.

Post-transcriptional regulation of *RCN2* has been investigated in mechanistic detail in the context of the *DUXAP8*–miR-1297 axis. In cervical cancer cells, wild-type and binding-site-mutant reporter assays supported site-specific targeting of the *RCN2* 3′ UTR by miR-1297, while Argonaute 2 (Ago2)-associated RNA immunoprecipitation and expression-perturbation experiments further supported miR-1297-dependent regulation of *RCN2*. Complementary reporter and RNA pull-down assays supported the *DUXAP8*–miR-1297 interaction; *DUXAP8* knockdown increased miR-1297 and reduced *RCN2* expression, whereas miR-1297 inhibition or ectopic *RCN2* overexpression counteracted multiple phenotypic effects of *DUXAP8* depletion, supporting a context-specific *DUXAP8*–miR-1297–*RCN2* competing endogenous-RNA (ceRNA) model in the cervical-cancer models examined [23].

In primary goat endometrial epithelial cells, 3′-UTR reporter assays supported miR-26a targeting of *RCN2*, but discordant *RCN2* mRNA and protein responses complicated the proposed circular RNA 3669 (circRNA3669)–miR-26a–*RCN2* ceRNA model [56]. A further inhibitory post-transcriptional relationship involving *RCN2* was reported for miR-155-5p in human aortic smooth muscle cells. In this system, miR-155-5p was reported to directly target *RCN2* and to reduce its mRNA expression within an RCN2–STAT3–miR-155-5p positive-feedback loop, whereas ectopic *RCN2* overexpression partially offset the miR-155-5p-mediated inhibition of high-phosphate-induced human aortic smooth muscle cell calcification [57]. A separate study in pituitary adenoma cells reported that *LINC01004* enhanced *RCN2* expression by sponging miR-323a-3p and miR-136-5p; this relationship was supported by in vitro mechanistic and functional assays, together with in vivo tumor-growth evidence consistent with the proposed axis. The resulting *LINC01004*–miR-323a-3p/miR-136-5p–*RCN2* ceRNA model should therefore be regarded as context-specific and provisional pending independent replication; the authors also noted that LINC01004 may regulate RCN2 through additional mechanisms beyond the ceRNA network that were not examined in the study [58]. RCN2 abundance can also be regulated post-translationally. In pancreatic cancer-associated fibroblasts, *MFAP5* knockdown predominantly reduced RCN2 protein abundance, with little corresponding change in *RCN2* mRNA. Cycloheximide-based protein-stability, ubiquitination, and proteasome-inhibition experiments indicated that MFAP5 stabilizes RCN2 by limiting its ubiquitin–proteasome-dependent degradation [50]. MFAP5 is therefore best classified as a post-translational stabilizer of RCN2 rather than as a transcriptional regulator.

Inflammatory and mechanical stimuli provide additional layers of context-dependent upstream regulation. In human umbilical vein endothelial cells, lipopolysaccharide (LPS) increased SP1 and RCN2 expression, while SP1-inhibition and RCN2-*overexpression* rescue experiments supported an LPS–SP1–RCN2 regulatory sequence in the inflammatory setting examined [43]. An exploratory proteomic study detected an RCN2 protein spot only after human cerebral endothelial cells were exposed to sera from patients with active relapsing–remitting multiple sclerosis (MS). Interferon beta-1b (IFN-β1b) treatment reduced the spot intensity by 66% compared with the MS-serum condition [59]. In primary mouse bone marrow macrophages and RAW264.7 macrophages, mechanical loading increased *Rcn2* expression and the study also documented mechanically induced RCN2 secretion in vitro and in vivo. *Piezo1* deletion in RAW264.7 macrophages reduced *Rcn2* expression, supporting the involvement of PIEZO1 in this mechanosensitive response; however, the downstream mechanism linking PIEZO1 to *Rcn2* expression, including any intervening transcriptional regulators, was not resolved [19]. Excessive mechanical loading increased *Rcn2* mRNA and RCN2 protein abundance in primary mouse chondrocytes, and RCN2 was also elevated in a human osteoarthritic-cartilage dataset and in anterior cruciate ligament transection (ACLT)-induced mouse osteoarthritis. *Piezo1* knockdown attenuated the loading-induced increase in *Rcn2*, but the downstream transcriptional mechanism linking PIEZO1 to *Rcn2* expression remained unresolved [51]. These studies suggest that RCN2 is mechanosensitive in multiple bone- and cartilage-associated cellular contexts, although the downstream regulatory mechanisms remain incompletely resolved and may differ across cellular contexts.

Metabolic and vascular conditions have likewise been associated with changes in RCN2 expression. Proteomic analysis of membrane-enriched fractions from the NES2Y human pancreatic β-cell line, followed by Western blot validation, showed that stearate-induced apoptosis was accompanied by significantly reduced RCN2 protein abundance. RCN2 abundance was also significantly reduced in whole-cell lysates, and oleate co-treatment counteracted both reductions [60]. Under high-glucose calcifying conditions, RCN2 expression was increased in human aortic vascular smooth muscle cells and was associated with enhanced osteogenic differentiation and calcification [61]. In the context of hyperphosphatemia, RCN2 expression was increased in calcified femoropopliteal arterial plaques from patients with chronic kidney disease and in a high-phosphate ex vivo aortic-ring culture model. However, a direct phosphate-responsive mechanism at the *RCN2* promoter was not established [57]. Conversely, high-density lipoprotein treatment significantly reduced *Rcn2* mRNA in primary mouse aortic endothelial cells, supporting context-specific negative regulation at the transcript level. Serum potassium was inversely associated with circulating RCN2 in patients with coronary artery disease, but KCl did not significantly alter endothelial *RCN2* mRNA, indicating that extracellular potassium remains a clinical correlate rather than a confirmed upstream regulator [62]. Current evidence indicates that RCN2 abundance is regulated through transcriptional, post-transcriptional, post-translational, and stimulus-responsive mechanisms whose relative contributions appear to vary across cellular and disease contexts.

### 5.3. Downstream Signaling and Regulatory Associations

Current evidence does not position RCN2 as an established core component of a single canonical signaling pathway. Instead, RCN2 appears to modulate distinct kinase-dependent, calcium-handling, protein-stability, inflammatory, and metabolic programs in a highly context-dependent manner. The mechanistic depth of these reported associations varies substantially: some are supported by physical-association studies, genetic or pharmacological perturbation, rescue experiments, and pathway inhibition, whereas others are based primarily on changes in phosphorylation, gene expression, or cellular phenotype following RCN2 manipulation. Moreover, signaling associated with intracellular full-length RCN2, including its canonical ER-associated form, should be distinguished from pathways initiated by extracellular RCN2 and from those mediated by Raptin, a cleaved RCN2-derived product.

Several of the most extensively characterized downstream pathways have been identified in cancer models. In HCC cells, RCN2 interacted with EGFR, while *RCN2* loss impaired EGF-induced EGFR dimerization, internalization, and phosphorylation, together with downstream ERK activation, cyclin D1 expression, and G1/S cell-cycle progression. AKT phosphorylation was not detectably altered under the conditions examined, indicating that the reported HCC mechanism is more appropriately described as an RCN2–EGFR–ERK–cyclin D1 axis than as generalized activation of both ERK and AKT signaling [18]. A mechanistically distinct pathway was identified in ESCC, where RCN2 directly bound PPP2CA and facilitated its UBR5-dependent ubiquitination and degradation through the homologous to the E6AP carboxyl terminus (HECT) domain of UBR5, thereby activating PI3K–AKT signaling, predominantly through AKT Ser473 phosphorylation [24]. Functional perturbation and rescue experiments linked the RCN2–PPP2CA–PI3K–AKT axis to metastatic behavior and cisplatin resistance, while *RCN2* suppression synergized with cisplatin to inhibit tumor growth and metastasis in subcutaneous and lung-metastasis models. Nevertheless, this mechanism should remain restricted to the ESCC context until independently demonstrated in other systems [24]. In oral squamous cell carcinoma (OSCC) cells, *RCN2* overexpression increased PKA activity, whereas *RCN2* knockout reduced it; inhibition of PKA with H89 reduced proliferation in *RCN2*-overexpressing cells, and AKT–mTORC signaling was identified as a downstream pathway through which PKA promoted proliferation [63]. These findings support a PKA-dependent AKT–mTORC pathway in the studied OSCC cell models but do not establish direct binding between RCN2 and PKA or fully resolve the molecular steps linking RCN2 to PKA activation.

RCN2 also contributes to a multistep stromal signaling program in pancreatic ductal adenocarcinoma (PDAC). In CAFs, MFAP5 stabilized RCN2 by limiting its ubiquitination-mediated proteasomal degradation, thereby sustaining ERK–STAT1 signaling and increasing STAT1-dependent *HAS2* and *CXCL10* expression [50]. Functionally, this stromal program was linked to extracellular-matrix remodeling and altered immune-cell infiltration. Mechanistically, *HAS2* and *CXCL10* are downstream transcriptional outputs of the MFAP5–RCN2–ERK–STAT1 pathway, whereas changes in tumor-cell programmed death-ligand 1 (PD-L1) occurred through subsequent CXCL10–CXCR3–AKT/mTOR–NF-κB signaling. Accordingly, HAS2, CXCL10, and PD-L1 should not be interpreted as direct RCN2-binding partners or as direct promoter-level targets of RCN2 [50].

Not all downstream effects of RCN2 are mediated through conventional phosphorylation cascades. In HCC cells, RCN2 increased MYC protein stability by reducing its ubiquitination and proteasomal degradation [42]. Cycloheximide-chase, ubiquitination, and proteasome-inhibition experiments supported this protein-stability effect. Several deubiquitinases with previously reported roles in MYC stabilization correlated with RCN2 expression in the same study, but their participation in the RCN2–MYC mechanism remains hypothetical because they were not directly perturbed [42]. PIGX was shown to interact with RCN1 and RCN2 and to function as a core component of an RCN1–PIGX–RCN2 complex; knockdown of *PIGX*, *RCN1*, or *RCN2* suppressed breast-cancer-cell growth, while knockdown of each component in BT-549 cells increased *EHD2* and *ZIC1* expression, supporting a model in which the complex promotes proliferation, at least in part, by repressing these putative tumor suppressors [53]. The intervening transcriptional mechanism was not resolved, and EHD2 and ZIC1 should not be interpreted as proteins that directly bind RCN2 or as genes directly regulated by RCN2 at the promoter level.

Calcium handling and mitochondrial apoptosis represent another mechanistically important group of downstream effects. In NPC cells, endogenous co-immunoprecipitation supported a cellular association between RCN2 and calreticulin; the authors proposed that this association may favor ER calcium retention and thereby limit stress-dependent mitochondrial Ca^2+^ loading [20]. Under cellular stress, *RCN2* knockout increased mitochondrial Ca^2+^ accumulation, mitochondrial-permeability-transition-pore opening, loss of mitochondrial membrane potential, and activation of cytochrome-c/Apaf-1/caspase-9-associated intrinsic apoptotic signaling, whereas *RCN2* overexpression generally attenuated these changes. These findings are consistent with an RCN2–CALR-associated model in which RCN2 limits stress-dependent mitochondrial Ca^2+^ loading and apoptosis; however, the study did not determine whether CALR is functionally required for these effects, demonstrate purified binary binding between RCN2 and CALR, or define the molecular interface linking their association to Ca^2+^ transfer [20]. More recently, in senescent human dermal fibroblasts, KB3409 reduced mitochondrial Ca^2+^ overload and mPTP opening, whereas *RCN2* knockdown abolished the additional Ca^2+^-lowering and mPTP-suppressive effects of KB3409 and prevented its increases in basal and ATP-linked respiration [64]. Notably, *RCN2* depletion itself reduced mitochondrial Ca^2+^ in this model, contrasting with the increase observed after *RCN2* loss in stressed NPC cells and further highlighting context-dependent RCN2-associated Ca^2+^ regulation [20,64]. The possible involvement of RCN2 in the STIM1–ORAI1 complex and store-operated Ca^2+^ entry is examined further in Section 6.1 [27].

STAT3-associated regulation has been reported in more than one disease context, but the underlying relationships are not identical. In human aortic smooth muscle cells exposed to high-phosphate conditions, RCN2 promoted STAT3 phosphorylation and participated in an RCN2–STAT3–miR-155-5p positive-feedback loop that supported osteogenic differentiation and vascular calcification [57]. Additionally, miR-155-5p negatively regulates *RCN2* mRNA expression within this loop [57]; therefore, the axis should be interpreted as a feedback-regulated RCN2–STAT3–miR-155-5p loop rather than as a simple linear RCN2-to-STAT3 pathway. In primary mouse chondrocytes, RCN2 physically associated with STAT3, and RCN2-dependent downstream STAT3 signaling was supported by increased STAT3 Tyr705 phosphorylation after *Rcn2* overexpression and by attenuation of excessive-mechanical-loading-induced STAT3 phosphorylation and nuclear translocation after *Rcn2* knockdown [51]. *Rcn2* perturbation linked this signaling event to osteoarthritis-associated chondrocyte responses, including reduced *Col2a1* and increased *Mmp13* and *Adamts5* expression, and the STAT3 inhibitor Stattic attenuated the abnormal chondrocyte metabolism exacerbated by *Rcn2* overexpression under excessive mechanical loading [51]. RCN2 was also associated with an osteogenic phenotypic switch in human aortic vascular smooth muscle-cell models under diabetic/calcifying conditions, with increased RUNX2 and Osterix, reduced α-smooth muscle actin (α-SMA) and smooth muscle protein 22α (SM22α), and enhanced mineralization [61].

RCN2-dependent downstream inflammatory signaling and vascular regulatory outputs have been described in endothelial systems. In LPS-treated human umbilical vein endothelial cells, RCN2 promoted reactive-oxygen-species (ROS) accumulation and downstream NLRP3–caspase-1–GSDMD-associated pyroptotic signaling, accompanied by increased LDH and interleukin-1β (IL-1β) release, while antioxidant treatment with NAC attenuated the observed responses [43]. *RCN2* knockdown also counteracted the LPS-mediated reduction in eNOS phosphorylation in this inflammatory model, suggesting a ROS-associated effect on eNOS signaling; however, the study did not demonstrate a physical interaction between RCN2 and eNOS [43]. In mouse aortic endothelial cells, *Rcn2* knockdown reduced basal and oxPAPC-induced VCAM-1 and MCP-1 production, supporting a role for RCN2 in endothelial inflammatory activation; however, the intervening signaling mechanism was not experimentally defined [65]. A separate cardiovascular study linked *Rcn2* loss to lower basal blood pressure and attenuated angiotensin II (ANG II)-induced hypertension in mice. In endothelial cells, *Rcn2* knockdown increased nitrite/nitrate levels as indices of nitric oxide (NO) production, whereas carotid arteries from *Rcn2*−/− mice exhibited enhanced NO-mediated vasorelaxation without increased eNOS expression, consistent with greater endothelial NO bioavailability [66]. This endothelial eNOS/NO-related cardiovascular mechanism should be distinguished from the acute LPS–ROS–pyroptosis model, although both studies implicate RCN2 in endothelial eNOS/NO-associated regulation [43,66].

Beyond its canonical ER-luminal localization, extracellularly available RCN2 has been reported to exert cell-extrinsic effects in several experimental contexts. An early cell-treatment study showed that exogenous RCN2 reduced intracellular and secreted collagen type I protein levels in human cardiac fibroblasts and decreased collagen type III and fibronectin protein levels in a dose-dependent manner [67]. In bone-marrow adipocyte models, extracellular RCN2 increased cAMP production, activated PKA, and promoted HSL- and ATGL-associated lipolytic responses. Knockdown of either NRP2 or ITGβ1 impaired these responses, supporting receptor-complex-dependent cAMP–PKA signaling [19]. In the same study, recombinant RCN2 increased the expression of fatty acid oxidation (FAO)-associated genes, including *Cpt1b*, *Cpt2*, *Acadm*, and *Acadl*, in bone marrow stromal cells (BMSCs) and promoted osteogenic outputs, including *Runx2*/*Sp7* expression and mineralization [19]. A recent study showed that RCN2 promoted calcification and inflammatory responses in cartilage endplate (CEP) cells through activation of intracellular cAMP–PKA signaling in an in vitro inflammatory model. The study further identified exosome-associated extracellular RCN2 as a mediator that activated the same signaling pathway and induced calcification in recipient cells [22]. A related but conceptually distinct extracellular signaling mechanism is mediated by Raptin, a cleaved and secreted hormone derived from RCN2 rather than canonical full-length RCN2. Raptin bound GRM3 on neurons in the hypothalamic paraventricular nucleus and stomach and activated downstream PI3K–AKT signaling. The study further showed that Raptin–GRM3 signaling promoted kinesin heavy chain (KHC)-dependent mitochondrial transport and mitochondrial ATP production required for neuronal activation and, in vivo, suppressed food intake and gastric emptying [21].

Reproductive, developmental, and neurocognitive studies further illustrate the context dependence of RCN2-associated signaling. In primary goat endometrial epithelial cells, RCN2 promoted proliferation and suppressed apoptosis, while knockdown reversed these effects; phosphorylation changes implicated PI3K–AKT–mTOR and JNK/P38 signaling [56]. In mouse embryonic stem cells, *Rcn2* overexpression activated ERK1/2 and promoted extraembryonic-endoderm differentiation, whereas *Rcn2* knockdown substantially attenuated *Stk40*-induced ERK1/2 phosphorylation and prevented *Stk40*-induced differentiation, supporting a functional requirement for RCN2 downstream of STK40. Notably, RCN2 activated ERK1/2 without activating RAS, whereas STK40 activated both, arguing against a simple linear STK40–RCN2–RAS cascade and leaving the precise signaling relationship mechanistically unresolved [48]. A subsequent study of mouse preimplantation embryos showed that *Stk40* silencing increased *Rcn2* mRNA and reduced blastocyst formation, without establishing a causal role for *Rcn2* in the developmental phenotype [68]. In mouse hippocampal HT22 neuronal cells, *Rcn2* knockdown reduced synaptophysin (Syn) abundance and increased total glycogen synthase kinase 3β (GSK3β) and phosphorylated Tau levels, whereas *Rcn2* overexpression produced the opposite pattern and partially reversed the corresponding high-glucose-induced changes, consistent with attenuation of downstream GSK3β–Tau-associated signaling. In vivo, hippocampal *Rcn2* overexpression increased Syn and neuronal nuclei (NeuN) signals and partially improved cognitive performance in db/db mice, whereas conditional hippocampal *Rcn2* deletion reduced these markers and impaired cognitive performance [69].

Current evidence positions RCN2 as a context-dependent modulator of diverse downstream signaling and regulatory programs rather than as a core component of a single conserved signaling cascade. Accordingly, these reported relationships should be interpreted in light of the type and strength of the underlying evidence, with careful attention to the RCN2 molecular form involved, its intracellular or extracellular localization, and the species, tissue, cell type, and experimental system in which each relationship was observed.

## 6. Pathobiological Contexts of RCN2

RCN2 biology extends from its foundational identity as an ER-associated Ca^2+^-binding protein to later context-specific roles in stress-responsive mitochondrial apoptosis and extracellular signaling [15,19,20,22]. Within disease-related studies, current evidence does not indicate a single conserved RCN2 pathway; reported effects instead vary across cancer-related and non-malignant contexts, cell types, species, and experimental systems [18,24,50,51,66]. Pivotal primary RCN2 studies, organized by biological and disease context and classified by evidence type, are summarized in Table 1.

### 6.1. Calcium, Stress Adaptation, and Organelle-Linked Contexts

Given its canonical ER-associated EF-hand architecture, RCN2 has long been linked to Ca^2+^-dependent ER functions; however, direct regulation of defined calcium channels or ER-to-mitochondria Ca^2+^-transfer machinery remains unproven [11,12,15]. Importantly, the original study argued that the relatively low abundance of ERC-55 made it unlikely to function as a major ER Ca^2+^-storage protein and proposed instead that it might regulate Ca^2+^-dependent activities in the ER by modulating the function of other proteins [15].

Among the available disease models, NPC currently provides the strongest RCN2-directed evidence linking ER-associated calcium handling to stress-responsive mitochondrial apoptosis, although CALR dependency and the mechanism of ER-to-mitochondria Ca^2+^ transfer remain unresolved [20]. By contrast, in senescent human dermal fibroblasts, *RCN2* knockdown reduced mitochondrial Ca^2+^ accumulation, providing an opposite RCN2-directed phenotype to that observed in stressed NPC cells and further supporting context-dependent RCN2-associated organelle Ca^2+^ regulation [20,64]. In an independent HEK-293 model, inducible calreticulin expression increased free ER-luminal Ca^2+^ and ER refilling while altering mitochondrial Ca^2+^ responses and reducing mitochondrial membrane potential, providing mechanistic context but not independent validation of an RCN2-dependent pathway [74].

Additional calcium-related evidence remains preliminary. In HEK293 cells overexpressing STIM1 and ORAI1, RCN2 was recovered from a tandem-affinity-purified STIM1–ORAI1 complex, colocalized with STIM1 in the resting ER, and redistributed into collar-like structures surrounding STIM1 clusters after thapsigargin-induced store depletion. However, an RCN2 construct in which all six EF-hand motifs were mutated produced only a slight reduction in the peak store-operated Ca^2+^ response measured by single-cell Ca^2+^ imaging [27]. An independent engineered ascorbate peroxidase 2 (APEX2)-based proximity-proteomic study likewise detected RCN2 among proteins located near STIM1 before store depletion, providing limited orthogonal support for basal spatial proximity but not for direct binding or functional participation in store-operated Ca^2+^ entry [75]. These findings position RCN2 as a candidate STIM1-associated, ER-proximal factor rather than a defined core component of store-operated Ca^2+^ entry.

A separate line of evidence links RCN2 to stimulus-dependent ER stress responses and candidate ER proteostasis contexts. In Huh7 hepatoma cells, thapsigargin-induced ER Ca^2+^-store depletion was accompanied by a pronounced time-dependent decrease in RCN2 protein and reduced *RCN2* mRNA, whereas RCN2 protein increased slightly after prolonged MG132 treatment and more strongly following dithiothreitol (DTT) exposure, indicating differential regulation across distinct ER-stress paradigms without identifying the responsible regulatory mechanism or UPR branch [76]. In a comparative SILAC–IP–MS/MS analysis of wild-type and misfolding-prone α1(A322D)-containing GABA_A_ receptors in HEK293T cells, RCN2 was identified among the ER-localized candidate interactors shared by both receptor interactomes, although the association and its functional relevance were not independently validated [77]. These observations broaden the organelle-linked context of RCN2 by encompassing both ER Ca^2+^-store perturbation and a candidate association with GABA_A_-receptor biogenesis in the ER.

### 6.2. RCN2 in Cancer-Related Contexts

The available cancer literature collectively supports RCN2 as a context-dependent tumor-associated factor rather than as an established oncogenic driver across malignancies. RCN2-focused studies have linked its expression or function to tumor growth, malignant cellular behavior, apoptosis resistance, stromal remodeling, metastasis, and treatment resistance in specific tumor settings. By contrast, transcriptomic, proteomic, and secretomic studies in which RCN2 appears as one component of a broader molecular signature should be regarded as discovery-level evidence unless supported by independent RCN2-specific clinical or functional validation. The principal study-level evidence across cancer contexts is summarized in Table 1.

HCC and colorectal cancer (CRC) were among the earliest epithelial tumor settings in which RCN2 was evaluated using clinical specimens and functional models. In HCC, independent studies associated increased RCN2 expression with adverse clinicopathological features and supported its contribution to tumor growth and invasive behavior through context-specific mechanisms [18,42]. In CRC, increased tumor RCN2 was associated with tumor size, invasion depth, and unfavorable disease-free and overall survival, while *RCN2* knockdown reduced cellular proliferation and xenograft growth [70]. In contrast, a CREC-focused protein-expression study found comparable ERC-55 (RCN2) levels in normal-derived NCM460 cells and the colorectal cancer cell lines SW480 and HCT116; ERC-55 was therefore not further examined in the patient biopsies [78]. A subsequent CREC-family analysis independently associated higher RCN2 expression with poorer overall and disease-free survival and bioinformatically inferred immune-cell infiltration [79]. A later HCC multi-omics analysis incorporated RCN2 into a programmed-cell-death/metabolism prognostic framework but did not include RCN2-directed functional validation [80].

ESCC, NPC, and OSCC extend the cancer-related evidence for RCN2 to metastatic progression, treatment resistance, stress-responsive tumor-cell survival, and adverse clinicopathological behavior. In ESCC, increased RCN2 expression was associated with metastatic progression and worse survival, while RCN2 suppression reduced metastatic behavior and enhanced cisplatin responsiveness in preclinical models [24]. In NPC, RCN2 promoted tumor progression in association with reduced stress-induced mitochondrial apoptosis [20]. In OSCC, independent studies associated increased RCN2 expression with lymph-node metastasis, advanced tumor–node–metastasis (TNM) stage, poorer prognosis, and functional promotion of proliferation, motility, invasion, and tumor progression [63,71].

Regulatory RNA-mediated studies further broaden the cancer contexts of RCN2, although their evidential strength differs. Functional and rescue experiments supported *DUXAP8*–miR-1297-dependent *RCN2* upregulation in cervical cancer [23] and *LINC01004*–miR-323a-3p/miR-136-5p-dependent *RCN2* regulation in pituitary adenoma models [58]. By contrast, the proposed *THAP7-AS1*/hsa-miR-155-5p/*RCN2* network in cholangiocarcinoma was derived from The Cancer Genome Atlas (TCGA)-based computational analysis without RCN2-directed functional validation and should remain discovery-level evidence [81].

RCN2 also illustrates a non-tumor-cell-autonomous cancer context. This perspective is particularly relevant to PDAC, a CAF-rich desmoplastic tumor in which stromal remodeling and CAF–tumor crosstalk influence disease progression and therapeutic response [82]. Earlier MFAP5-targeting work linked stromal MFAP5 blockade to reduced fibrosis and enhanced chemosensitivity in ovarian and pancreatic cancer models [83]. Within this stromal framework, Duan et al. identified RCN2 as part of an MFAP5-dependent CAF axis with treatment-relevant implications for remodeling the PDAC microenvironment [50]. This positions PDAC as an RCN2 context that is primarily stromal/tumor microenvironment-associated rather than tumor-cell-autonomous.

Breast and mammary cancer-related evidence expands the RCN2 cancer spectrum, but remains less definitive than the RCN2-focused functional evidence described above. In breast-cancer models, Nakakido et al. linked RCN2 to cancer-cell growth in the context of a PIGX-centered RCN1/PIGX/RCN2 complex, with knockdown of complex components suppressing cell growth and increasing expression of putative tumor suppressors such as *EHD2* and *ZIC1* [53]. However, this evidence supports a cancer-relevant RCN2-containing complex rather than a fully resolved RCN2-specific oncogenic mechanism in breast cancer. Additional mammary/breast cancer studies identified RCN2/ERC-55 in immunogenomic or proteomic marker-discovery contexts, supporting only preliminary relevance rather than causal RCN2-dependent tumor biology [26,84].

Discovery-level observations have also been reported in several additional malignancies. Proteomic analysis of seven BRAF^V600E-mutant papillary thyroid carcinomas identified an RCN2 protein spot with 2.48-fold higher abundance than in matched normal thyroid tissues, without independent RCN2 validation [85]. A cDNA microarray screen reported lower *RCN2* expression in 5-fluorouracil- and cisplatin-resistant SNU638 gastric cancer sublines than in parental SNU638 cells [86]. In prostate cancer, molecular cytogenetic analysis of the LNCaP cell line mapped the t(1;15)(p22;q22) rearrangement to a chromosome 15 breakpoint with loss of material containing *RCN2* and nearby genes; this cell-line observation should remain discovery-level evidence and should not be interpreted as proof of an RCN2-specific tumor-suppressor role [87]. RCN2 has also appeared in non-small-cell lung cancer gene-expression, ovarian-cancer drug-response proteomic, and primary central nervous system lymphoma exosomal-miRNA target-prediction studies; these observations should likewise be retained as discovery-level evidence rather than expanded into mechanistic claims [25,88,89]. A recent LUAD study reported increased expression of *RCN2*, alongside *RCN1*, *RCN3*, *SDF2*, and *CALU*, in LUAD cell lines relative to normal controls, with independent validation datasets confirming these findings at both transcript and protein levels; this evidence further supports a discovery-level link between RCN2 and LUAD [90]. An additional recent study in T24 bladder-cancer cells identified RCN2 in a NUPR1 IP–MS screen and provided co-immunoprecipitation support for an RCN2–NUPR1 cellular association, but did not include RCN2-directed functional validation [91].

The most persuasive evidence comes from tumor settings in which clinical associations are supported by RCN2-directed functional perturbation, whereas discovery-level and historical observations should remain hypothesis-generating. Future studies should define tumor-type-specific RCN2 dependencies, distinguish tumor-cell-autonomous from stromal RCN2 functions, and test their relevance to metastasis, treatment resistance, and tumor–microenvironment remodeling.

### 6.3. RCN2 in Non-Malignant Disease Contexts

Outside cancer, RCN2 has been examined in vascular, inflammatory, calcific, skeletal, metabolic, infectious, and neurocognitive disease-related contexts. Limited candidate-gene evidence has also appeared in ocular disease genetics, where *RCN2* was included in a negative sequencing screen of positional candidate genes within a refined 15q keratoconus-with-cataract linkage interval [92]. The vascular literature currently provides the broadest non-malignant disease evidence. In atherosclerosis-related mouse genetics, *Rcn2* was prioritized as a candidate gene within the Ath29 susceptibility locus and functionally linked to endothelial inflammatory-mediator regulation, providing an early genetic and inflammatory connection between RCN2 and atherosclerotic vascular disease biology [65]. In spontaneously hypertensive rats, *Rcn2* was prioritized as one of four cis-regulated candidate genes associated with coronary arteriolar medial area, but not with blood-pressure indices [93]. Subsequent vascular studies extended this relevance to blood-pressure regulation, endothelial dysfunction, atherosclerotic lesions, and vascular calcification. Loss of *Rcn2* lowered basal blood pressure and attenuated angiotensin II-induced hypertension in vivo, supporting a role for RCN2 in vascular functional regulation [66]. RCN2 was also increased in atherosclerotic lesions and in circulating samples from patients with coronary artery disease (CAD) or peripheral artery disease, although its biomarker implications are better considered in the clinical relevance section [62]. An ER-stress-focused CAD study identified *RCN2* within a LASSO-derived PBMC transcriptomic biomarker panel and confirmed lower *RCN2* expression in CAD PBMC samples by qRT-PCR [94]. In calcific vascular models, RCN2 promoted osteogenic differentiation and calcification of human aortic vascular smooth muscle cells under diabetic conditions and contributed to high-phosphate-induced calcification through an RCN2–STAT3–miR-155-5p feedback loop [57,61]. In calcific aortic valve stenosis, sex-stratified valve transcriptomics identified *RCN2* among calcification-associated transcripts overexpressed in stenotic valves from female patients [95]. In LPS-induced endothelial inflammatory injury, RCN2 was further implicated in endothelial pyroptosis, supporting its involvement in endothelial inflammatory stress responses [43].

Bone marrow, bone-regeneration, and cartilage studies extend non-malignant RCN2 biology into mechanically responsive and reparative musculoskeletal tissues. In bone marrow, mechanically induced macrophage secretion of RCN2 linked load-responsive signaling to marrow adipocyte lipolysis, osteogenesis, and lymphopoietic support, thereby extending RCN2 biology beyond its canonical intracellular ER context [19]. A recent calvarial-defect study reported that RCN2 deficiency impaired bone repair and aggravated inflammatory responses, whereas delivery of RCN2-modified M2 macrophage-derived exosomes supported immuno-osteogenic coupling and bone regeneration [72]. Quantitative proteomics of postnatal mouse femoral head cartilage showed that RCN2, together with RCN1 and calumenin, was significantly more abundant on postnatal day 21 (P21) than at postnatal day 3 (P3), consistent with a maturation-associated increase in ER-luminal calcium-binding proteins [96]. In a modular co-culture system comprising rabbit chondrocytes and rabbit bone mesenchymal stem cells, *RCN2* knockdown in chondrocytes increased nuclear factor of activated T cells 1 (NFAT1), type II collagen, and SRY-box transcription factor 9 (SOX9) expression, whereas exogenous RCN2 reduced NFAT1 expression, supporting a context-dependent role for RCN2 in NFAT-associated chondrocyte maturation [97]. In cartilage, excessive mechanical loading increased *Rcn2* expression in chondrocytes; *Rcn2* knockdown or chondrocyte-specific deletion alleviated ACLT-induced osteoarthritis progression, whereas *Rcn2* overexpression aggravated it, with experimental evidence linking RCN2 to chondrocyte responses and osteoarthritis progression [51]. Single-cell RNA-sequencing of bovine caudal intervertebral discs identified *RCN2* among novel nucleus-pulposus marker genes and ER/stress-associated transcripts [98]. A cartilage endplate study further reported that extracellular and exosome-associated RCN2 promoted CEP-cell calcification and inflammatory responses through cAMP–PKA signaling, suggesting a possible contribution to intervertebral disc degeneration-related pathology [22].

In the neurological and neurocognitive domain, peripheral expression studies have linked RCN2 to absence epilepsy with inconsistent strength. A monozygotic-twin lymphoblastoid-cell-line study reported differential *RCN2* expression that was reconfirmed by qRT-PCR in an independent absence-epilepsy cohort, whereas a later study in untreated patients found *RCN2* dysregulation in individual experiments but emphasized poor reproducibility across groups [99,100]. An early cDNA microarray screen reported 2.81-fold higher hippocampal *Rcn2* expression in 12-month-old male SAMP8 mice than in age-matched SAMR1 controls [101]. In a human multi-omics study, plasma RCN2 was positively associated with conversion from mild cognitive impairment to Alzheimer’s disease, whereas Mendelian randomization indicated an apolipoprotein E (*APOE*)-dependent reverse association from Alzheimer’s disease genetic liability to RCN2 levels [102]. In a rat model of gestational binge alcohol exposure, fetal cerebellar proteomics showed higher RCN2 abundance than in pair-fed controls, although the difference did not survive correction for multiple comparisons [103]. In a preterm-pig model of intra-amniotic prenatal LPS exposure, cerebrospinal fluid (CSF) proteomics detected reduced RCN2 at birth among proteins altered by prenatal inflammation [104]. A whole-genome sequencing study in the Army Study to Assess Risk and Resilience in Servicemembers (Army STARRS) cohort reported the uncommon nonsilent *RCN2* variant rs144252373/p.L57I among single-variant associations with suicide attempt [45]. More recently, Zhang et al. positioned hippocampal RCN2 within diabetes-associated cognitive dysfunction, supporting its inclusion as a non-malignant disease context without extending the claim to a primary neurodegenerative mechanism [69]. In primary human hepatocytes, productive hepatitis B virus infection reduced RCN2 protein abundance without a corresponding change in *RCN2* mRNA, whereas *RCN2* knockdown did not consistently affect viral RNA or antigen levels [73]. A cardiac-fibroblast proteomic study centered on RCN3 provides a limited RCN2-related fibrotic context, as recombinant RCN2 was evaluated alongside other reticulocalbins and reduced selected extracellular-matrix protein levels in human cardiac fibroblasts [67]. The Raptin study adds an endocrine-metabolic disease context by linking an RCN2-derived hypothalamic hormone to sleep-deficiency-associated obesity and night-eating syndrome [21]. The context-dependent relationships among RCN2 molecular form or state, compartment or localization state, biological context, and principal reported outcomes are synthesized in Figure 3.

**Figure 3:**
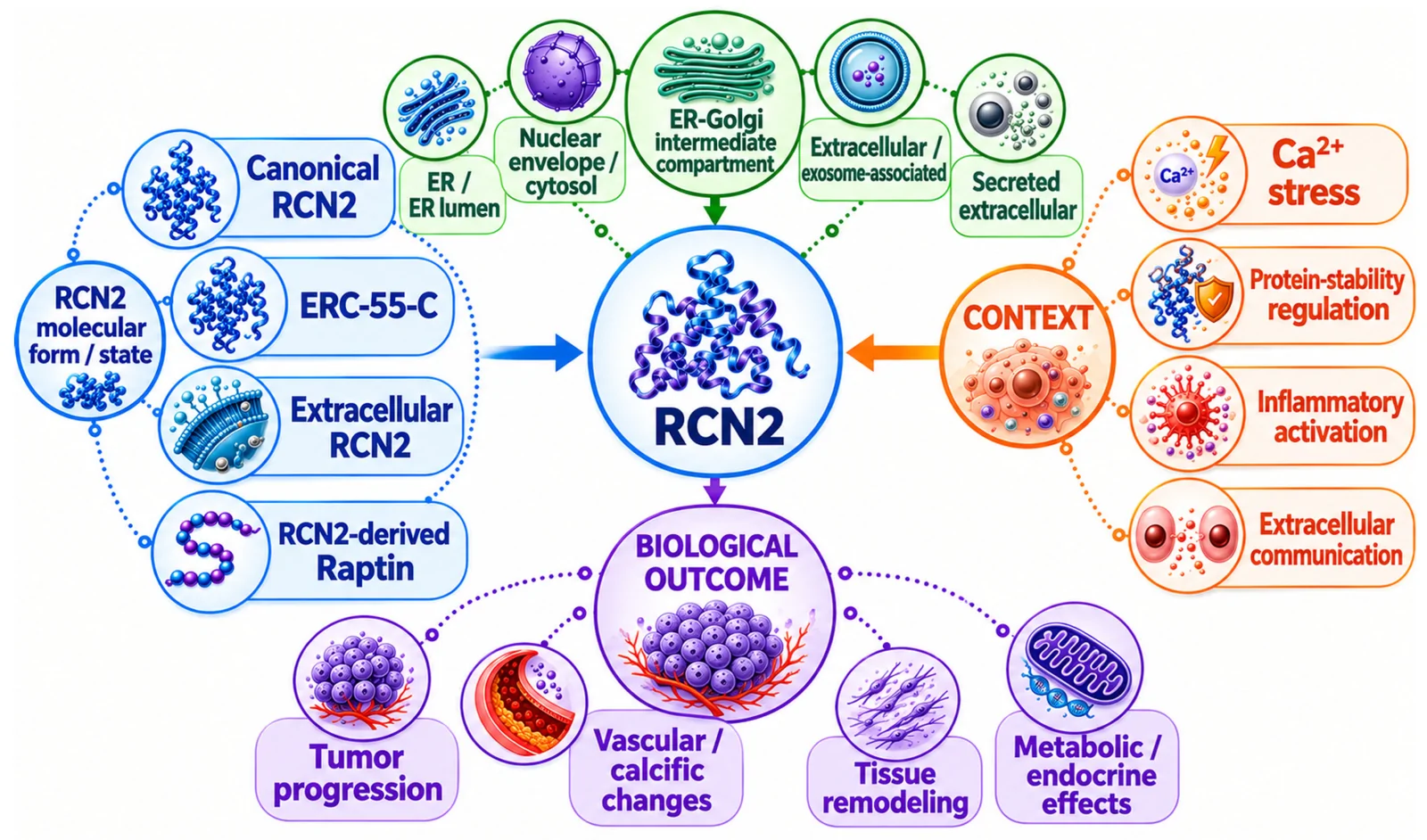
Context-dependent framework linking RCN2 molecular form/state, compartment/localization state, biological context, and outcome. This schematic integrates canonical RCN2, ERC-55-C, extracellular RCN2, and RCN2-derived Raptin with reported compartment/localization states, including the ER/ER lumen, nuclear envelope/cytosol, ER–Golgi intermediate compartment, extracellular/exosome-associated localization, and secreted extracellular states. Representative biological contexts include Ca^2+^/stress, protein-stability regulation, inflammatory activation, and extracellular communication, with reported outcomes including tumor progression, vascular/calcific changes, tissue remodeling, and metabolic/endocrine effects. The schematic is an evidence-informed conceptual synthesis and does not imply that every form/state–localization–context–outcome combination has been experimentally demonstrated. Abbreviations: ER, endoplasmic reticulum; RCN2, reticulocalbin-2.

## 7. Clinical Relevance and Translational Limitations of RCN2

The clinical relevance of RCN2 remains suggestive rather than established. Across the current literature, the strongest translational signals come from disease settings in which clinical expression, prognostic, or circulating evidence is available alongside RCN2-focused experimental data, but even these studies generally stop short of independent multicenter validation, standardized assay thresholds, or prospective clinical testing [18,24,62,63,70]. By contrast, database-driven, proteomic, secretomic, or single-context observations should remain hypothesis-generating and should not be used alone to define RCN2 as a validated biomarker or prognostic marker [25,26,79,84,88]. The context-specific translational evidence and principal limitations of RCN2 are summarized in Table 2.

From a biomarker perspective, the central unresolved issue is not whether RCN2 can vary with disease state, but whether it improves clinically relevant prediction beyond established variables. Future studies should therefore evaluate tissue RCN2 and, where appropriate, circulating RCN2 in disease-specific cohorts with predefined clinical endpoints, assay reproducibility, standardized cutoffs, and adjustment for conventional clinicopathological factors [62,63,70]. Prognostic interpretation will also require external validation, survival-specific endpoints, and multivariable modeling rather than reliance on unadjusted expression–outcome associations alone. Biomarker interpretation should further distinguish intracellular ER-associated RCN2, extracellular or exosome-associated RCN2, and the RCN2-derived hormone Raptin, because these entities may require different sampling and assay strategies and may not carry equivalent biological or clinical meaning [15,19,21,22].

Therapeutic translation is less mature. Existing studies support experimental targetability of RCN2-linked pathways or cellular contexts, but they do not yet establish RCN2 itself as a clinically actionable drug target [24,50,51,64,72]. Key unresolved issues include the druggability of the relevant RCN2 molecular form, tissue selectivity, safety of modulating a broadly expressed ER-associated protein, cell-type-specific localization in clinically relevant tissues, availability of on-target pharmacodynamic markers, and validation in disease models that better approximate clinical biology. At present, and in the absence of clinical trials testing RCN2-directed interventions, RCN2 should be framed as a candidate translational node whose biomarker, prognostic, and therapeutic value requires systematic validation rather than as a clinically validated marker or target. Accordingly, priority should be given to molecular-form-resolved assays, validation of endogenous RCN2 biology in disease-relevant cell types, and prospectively designed cohorts capable of testing whether RCN2 adds clinical value beyond established variables. Human/clinical and RCN2- or Raptin-directed functional evidence across disease contexts is summarized in Figure 4.

## 8. Methodological Limitations

As a structured narrative review, this work has methodological limitations that warrant acknowledgment. Study identification, eligibility assessment, and evidence appraisal were conducted by a single author without independent duplicate review. Given the structured narrative design, no review protocol was prospectively registered and no formal risk-of-bias instrument was applied. The evidence-category framework and translational labels used here were developed specifically to organize and interpret the heterogeneous RCN2 literature and should not be regarded as a validated evidence-grading system. In addition, although no formal language restriction was applied during the search, the evidentiary synthesis was limited to publications that could be evaluated reliably in English.

## 9. Conclusions

Collectively, the available evidence supports viewing RCN2 as a context-dependent modulator whose effects vary with molecular form and subcellular compartment, rather than as a component of a single conserved signaling pathway. Across the studies reviewed, RCN2 has been implicated in calcium- and stress-associated signaling, protein stability, inflammatory activation, extracellular communication, and tissue-specific disease programs. However, the strength of evidence varies substantially across biological contexts. The most persuasive disease evidence arises in settings where clinical or tissue-level associations are reinforced by RCN2-directed functional perturbation, whereas proteomic, transcriptomic, database-derived, and other single-context observations lacking RCN2-directed validation should remain primarily hypothesis-generating. Future progress will require molecular-form-resolved assays, endogenous validation of proposed interactions and mechanisms in disease-relevant cell types, independent clinical cohorts capable of defining biomarker performance, and disease-relevant models that test therapeutic relevance. Until these requirements are met, RCN2 should be regarded as a biologically informative and translationally plausible candidate node rather than as a clinically validated biomarker or therapeutic target.

## Figures and Tables

**Figure 1:**
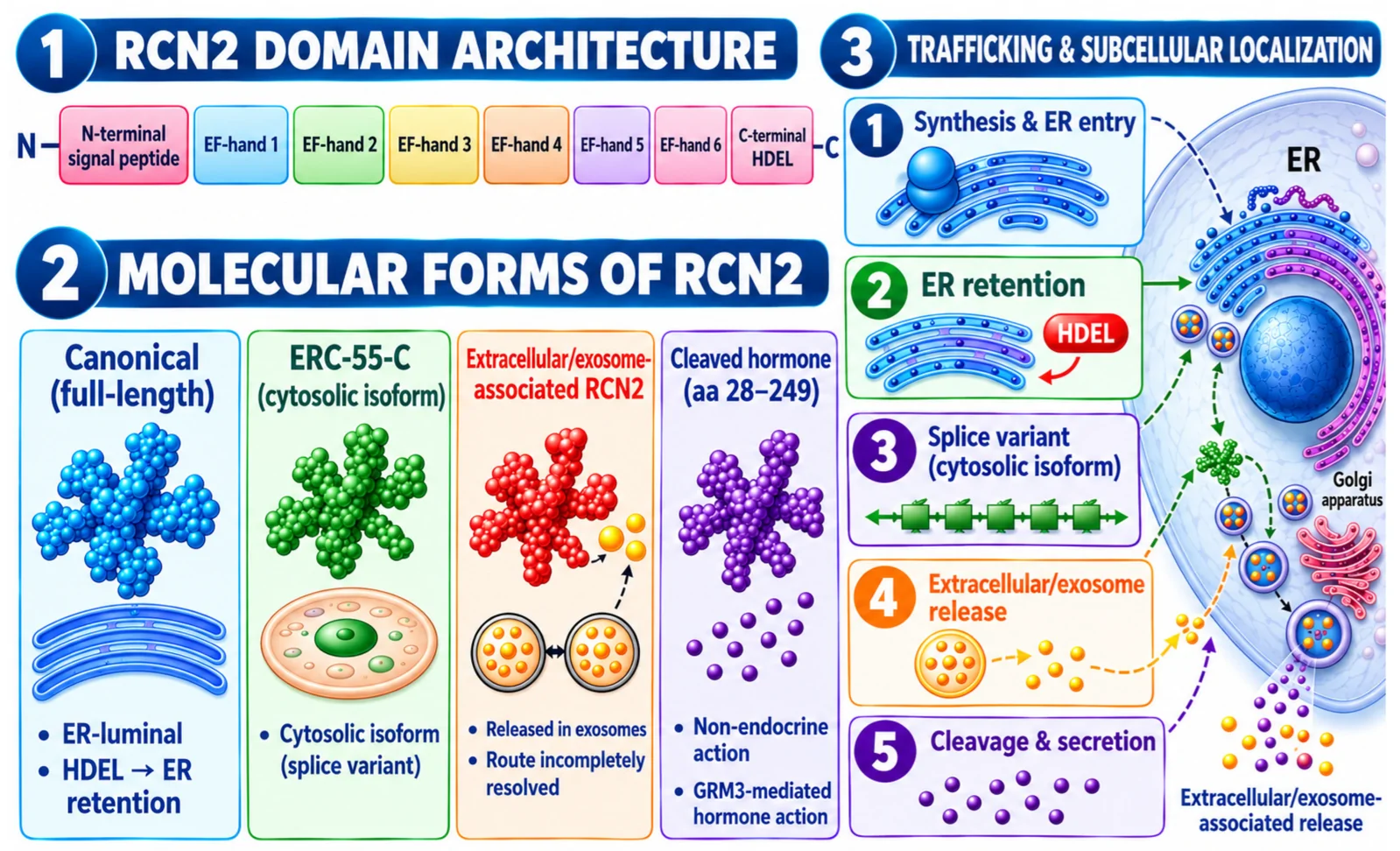
Molecular architecture, molecular forms, trafficking, and subcellular localization of RCN2. (1) Canonical RCN2 domain architecture, including an N-terminal signal peptide, six EF-hand motifs, and a C-terminal HDEL ER-retention/retrieval motif. The canonical precursor contains 317 amino acids and has an apparent molecular mass of approximately 55 kDa. (2) Principal RCN2 molecular forms and localization states represented in the current literature, including canonical ER-associated RCN2, the ERC-55-C cytosolic splice variant, context-specific extracellular/exosome-associated RCN2, and Raptin, a cleaved and secreted RCN2-derived hormone spanning residues 28–249. (3) Principal trafficking and localization contexts, including canonical ER entry and retention, splice-variant-associated cytosolic distribution, context-specific extracellular/exosome-associated release, and Raptin cleavage and secretion. The precise route(s) responsible for extracellular/exosome-associated RCN2 release remain incompletely resolved. This figure is a conceptual schematic based on information reported in the literature. Protein depictions are illustrative and do not represent experimentally determined or computationally predicted three-dimensional structures of RCN2 or its molecular forms. Abbreviations: aa, amino acids; ER, endoplasmic reticulum; HDEL, His-Asp-Glu-Leu; RCN2, reticulocalbin-2.

**Figure 4:**
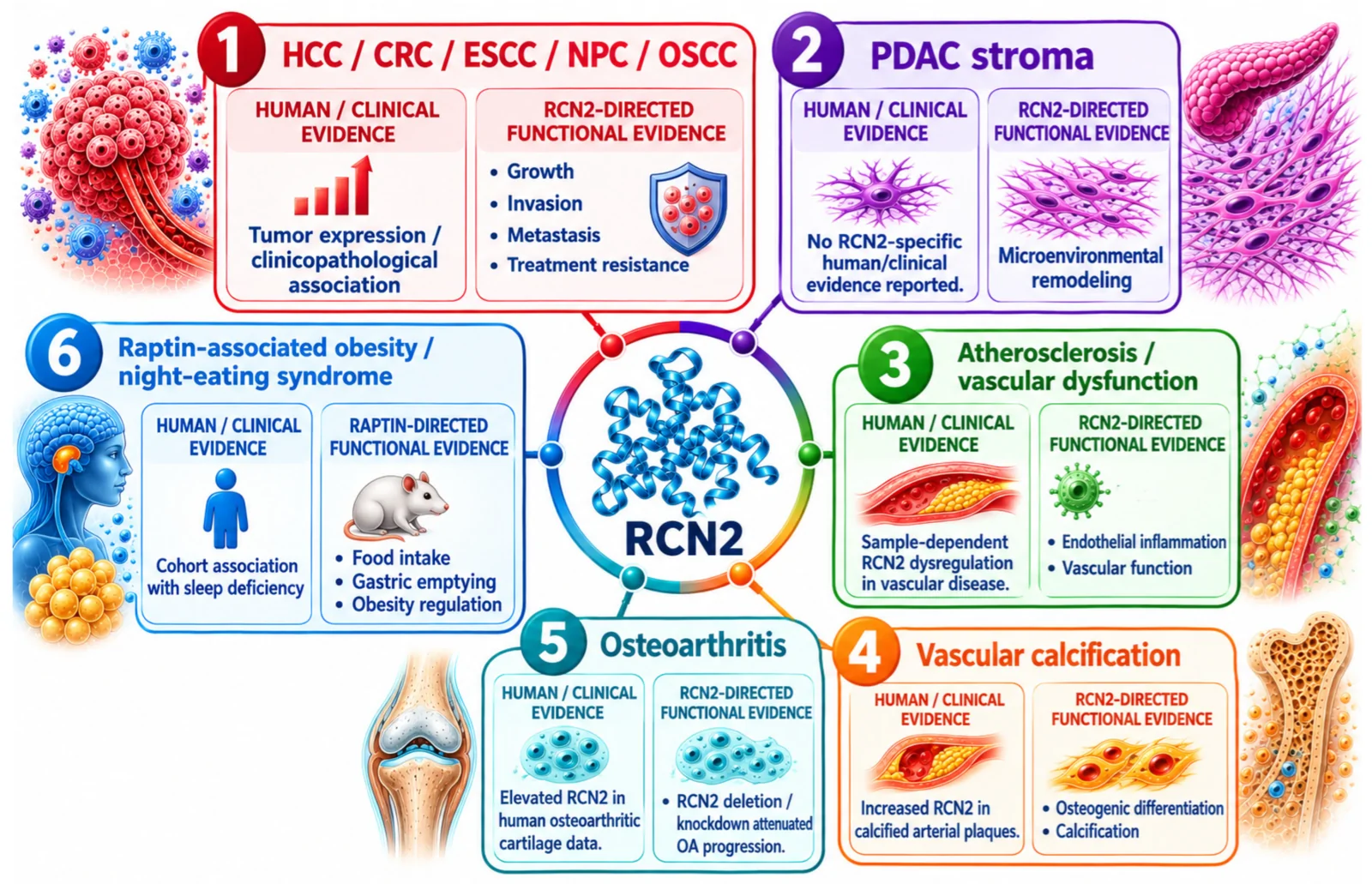
Evidence map of human/clinical and RCN2- or Raptin-directed functional evidence across disease contexts. The figure summarizes human/clinical and functional evidence across grouped epithelial cancers (HCC, CRC, ESCC, NPC, and OSCC), PDAC stroma, atherosclerosis/vascular dysfunction, vascular calcification, osteoarthritis, and Raptin-associated obesity/night-eating syndrome. Human/clinical and functional evidence are shown as parallel evidence streams, with Raptin-directed evidence distinguished from RCN2-directed evidence. In the grouped cancer panel, the listed functional outcomes are supported collectively across the represented tumor contexts and should not be interpreted as applying uniformly to every cancer type. Context-colored connectors identify disease categories and do not encode evidence strength or causal direction. Abbreviations: CRC, colorectal cancer; ESCC, esophageal squamous cell carcinoma; HCC, hepatocellular carcinoma; NPC, nasopharyngeal carcinoma; OA, osteoarthritis; OSCC, oral squamous cell carcinoma; PDAC, pancreatic ductal adenocarcinoma; RCN2, reticulocalbin-2.

**Table 1:** Pivotal primary RCN2 studies organized by biological and disease context.

Study	Biological or Disease Context	Experimental Model	Principal RCN2-Related Finding	Evidence Category
Molecular and cellular biology				
[15]	Foundational molecular biology	Recombinant human ERC-55; mammalian cells and tissues	Defined ER-localized, EF-hand Ca^2+^-binding ERC-55 and HDEL-dependent retention	Direct biochemical + functional
[33]	Gene identity and chromosomal localization	Human metaphase chromosomes; in situ hybridization	Linked ERC-55 to the *RCN2* gene; historical in situ hybridization assigned it to 15q22.33–q24.1, whereas current reference annotation places *RCN2* at 15q24.3	Foundational molecular/genetic
[17]	Isoforms and molecular diversity	Recombinant ERC-55; human tissues and cultured cells	Identified ERC-55 splice variants, cytosolic ERC-55-C, and direct in vitro partners	Direct biochemical
Protein interactions and signaling				
[16,34]	Papillomavirus E6 interaction	Yeast two-hybrid, GST assays, and mutational mapping	Demonstrated direct E6 binding and mapped an α-helical interaction motif in RCN2/ERC-55	Direct biochemical
[27] †	Store-operated Ca^2+^ entry	STIM1/ORAI1-overexpressing HEK293 cells	Recovered RCN2 within the STIM1–ORAI1 complex; functional effect on Ca^2+^ entry was modest	Cellular association/complex—preliminary
[64]	Cellular senescence and mitochondrial Ca^2+^ regulation	Senescent human dermal fibroblasts; *RCN2* knockdown and KB3409 treatment	Linked RCN2 to KB3409-dependent mitochondrial Ca^2+^, mPTP, and respiratory responses	Functional
[48]	Embryonic differentiation	Mouse embryonic stem cells; GST, co-IP, and perturbation assays	Linked direct STK40–RCN2 interaction to ERK1/2-associated ExEn differentiation	Direct biochemical + functional
[53]	PIGX-centered complex	293T and breast-cancer cell models	Identified an RCN1–PIGX–RCN2 complex supporting cancer-cell growth	Cellular association/complex + functional
[56]	Endometrial receptivity	Goat endometrium and primary GEECs	Supported miR-26a targeting of *RCN2* and linked RCN2 to proliferation, apoptosis, and PI3K–AKT–mTOR/JNK–P38 signaling	Functional
Cancer-related contexts				
[18,42]	Hepatocellular carcinoma	Patient tissues, HCC cells, and xenografts	RCN2 promoted proliferation, tumor growth, and invasive behavior through distinct mechanisms	Clinical observational + functional
[70]	Colorectal cancer	Paired tissues, CRC cells, and xenografts	Increased RCN2 expression accompanied tumor progression and supported proliferation and xenograft growth	Clinical observational + functional
[23]	Cervical cancer	Cell-based mechanistic assays and xenografts	*DUXAP8*–miR-1297-dependent *RCN2* upregulation promoted malignant phenotypes	Functional
[58]	Pituitary adenoma	Pituitary adenoma cells and xenograft models	*LINC01004*-mediated *RCN2* upregulation promoted malignant cellular phenotypes and tumor growth	Functional
[63,71]	Oral squamous cell carcinoma	Tumor tissues and OSCC cell models	Increased RCN2 supported proliferation, migration/invasion, and tumor progression	Clinical observational + functional
[50]	Pancreatic ductal adenocarcinoma stroma	CAFs, tumor cells, and mouse models	MFAP5-stabilized RCN2 supported matrix remodeling and altered immune-cell infiltration	Cellular association/complex + functional
[20]	Nasopharyngeal carcinoma	Patient tissues, NPC cells, and xenografts	RCN2 restrained stress-induced mitochondrial apoptosis and promoted tumor progression	Cellular association/complex + functional
[24]	Esophageal squamous cell carcinoma	Patient cohorts, ESCC cells, and mouse models	Direct PPP2CA binding linked RCN2 to metastasis and cisplatin resistance	Direct biochemical + functional
Non-malignant disease contexts				
[65]	Atherosclerosis and endothelial inflammation	Mouse genetics and aortic endothelial cells	Prioritized *Rcn2* at the Ath29 locus and linked it to inflammatory-mediator expression	Functional
[66]	Hypertension and endothelial function	*Rcn2*-null mice, isolated arteries, and endothelial cells	*Rcn2* loss lowered blood pressure and increased NO-dependent vasorelaxation	Functional
[57,61]	Vascular calcification	Human VSMCs, patient plaques, and ex-vivo aortic tissue	RCN2 promoted osteogenic conversion and calcification under diabetic or high-phosphate conditions	Clinical observational + functional
[43]	Endothelial inflammatory injury	LPS-treated HUVECs	RCN2 promoted ROS-associated endothelial pyroptotic responses	Functional
[51]	Osteoarthritis	Human datasets, mouse chondrocytes, and conditional mice	RCN2 promoted loading-induced STAT3 responses and osteoarthritis progression	Cellular association/complex + functional
[69]	Diabetes-associated cognitive dysfunction	HT22 neuronal cells, db/db mice, and conditional mouse models	Hippocampal RCN2 improved neuronal markers and cognitive performance	Functional
[72]	Bone regeneration	Macrophages, exosomes, and calvarial-defect mice	RCN2 deficiency impaired bone repair, whereas RCN2-modified M2 macrophage exosomes improved immuno-osteogenic coupling and regeneration	Functional
[73]	Hepatitis B virus infection	Primary human hepatocytes; proteomic, western-blot, and *RCN2*-knockdown	Productive HBV infection reduced RCN2 protein abundance without changing its mRNA; knockdown did not consistently affect HBV replication markers	Functional
Extracellular RCN2				
[19]	Mechanosensitive bone-marrow signaling	Macrophages, adipocytes, BMSCs, and mouse models	Secreted RCN2 acted through a functional receptor complex containing NRP2 and ITGβ1 and promoted lipolytic and osteogenic responses	Cellular association/complex + functional
[22]	Cartilage endplate degeneration	CEP cells and exosome-transfer models	Extracellular and exosome-associated RCN2 promoted calcification and inflammatory responses	Functional
RCN2-derived Raptin				
[21]	Appetite and obesity regulation	Neuronal and gastric systems, mouse models, and human cohorts	RCN2-derived Raptin bound GRM3 and suppressed feeding and gastric emptying	Direct biochemical + functional

† Zhan et al. (2008) [27] is not indexed in PubMed; an English-language full-text version was available and was appraised directly. Note: Evidence categories were defined as follows: direct biochemical, molecular characterization or binding demonstrated using biochemical or reconstituted assays; cellular association/complex, co-immunoprecipitation, complex capture, colocalization, or related cellular evidence without purified binary-binding confirmation; functional, RCN2-directed perturbation, rescue, pathway inhibition, or genetic models; clinical observational, associations in patient-derived tissues or cohorts; and foundational molecular/genetic, primary gene-identification, chromosomal-mapping, or molecular-characterization studies. Combined categories indicate support from more than one evidence type. In combined rows, findings summarize the collective evidence and do not imply that every listed study demonstrated each outcome. Abbreviations: AKT, protein kinase B; BMSC, bone marrow stromal cell; CAF, cancer-associated fibroblast; CEP, cartilage endplate; co-IP, co-immunoprecipitation; CRC, colorectal cancer; ER, endoplasmic reticulum; ERK, extracellular signal-regulated kinase; ESCC, esophageal squamous cell carcinoma; ExEn, extraembryonic endoderm; GEEC, goat endometrial epithelial cell; GRM3, metabotropic glutamate receptor 3; GST, glutathione S-transferase; HBV, hepatitis B virus; HCC, hepatocellular carcinoma; HDEL, His-Asp-Glu-Leu; HUVEC, human umbilical vein endothelial cell; ITGβ1, integrin β1; JNK, c-Jun N-terminal kinase; LPS, lipopolysaccharide; MFAP5, microfibril-associated protein 5; mTOR, mechanistic target of rapamycin; NO, nitric oxide; NPC, nasopharyngeal carcinoma; NRP2, neuropilin 2; ORAI1, calcium release-activated calcium channel protein 1; OSCC, oral squamous cell carcinoma; PIGX, phosphatidylinositol glycan anchor biosynthesis class X; PI3K, phosphoinositide 3-kinase; PPP2CA, protein phosphatase 2A catalytic subunit alpha; RCN1, reticulocalbin-1; RCN2, reticulocalbin-2; ROS, reactive oxygen species; STAT3, signal transducer and activator of transcription 3; STIM1, stromal interaction molecule 1; STK40, serine/threonine kinase 40; VSMC, vascular smooth muscle cell.

**Table 2:** Translational evidence appraisal of RCN2 across major disease contexts.

Disease Context	Human or Clinical Evidence	RCN2-Directed Functional Evidence	Current Translational Interpretation	Principal Limitation
HCC	Increased tumor *RCN2* expression associated with adverse clinicopathological features [18,42]	*RCN2* perturbation altered tumor growth [18] and invasiveness [42]	Tumor-associated expression with functional support	No external clinical validation
CRC	Higher tumor RCN2 associated with invasion depth and poorer survival [70], with independent survival support [79]	*RCN2* knockdown reduced proliferation and xenograft growth [70]	Candidate prognostic relevance	No standardized cutoffs or prospective validation
ESCC	Higher *RCN2* expression associated with metastatic progression and worse survival [24]	*RCN2* suppression reduced metastatic behavior and enhanced cisplatin response [24]	Preclinical treatment-resistance relevance	No independent clinical validation
NPC	Higher tumor *RCN2* expression was associated with advanced stage and poorer survival in NPC [20]	*RCN2* manipulation altered tumor progression and stress-induced mitochondrial apoptosis [20]	Mechanistic tumor-survival relevance	Clinical utility untested
OSCC	RCN2 overexpression associated with nodal metastasis and advanced stage [71], with poorer prognosis also reported [63]	*RCN2* perturbation altered invasiveness [71] and tumor progression [63]	Candidate prognostic relevance	No external prognostic validation
PDAC stroma	No RCN2-specific clinical evidence reported	RCN2-directed evidence supported stromal microenvironmental remodeling [50]	Preclinical stromal translational relevance	No RCN2-specific clinical validation
Atherosclerosis and vascular dysfunction	Increased lesion and circulating RCN2 contrasted with lower expression in CAD PBMCs [62,94]	*RCN2* perturbation altered endothelial inflammation and vascular function [65,66]	Context-dependent vascular biomarker signal	Discordant sample-dependent expression
Vascular calcification	Increased RCN2 in calcified arterial plaques [57]	*RCN2* perturbation promoted vascular calcification [57,61]	Human-associated and experimentally supported calcification relevance	No prospective clinical validation
Osteoarthritis	Elevated *RCN2* in human osteoarthritic-cartilage data [51]	*Rcn2* deletion or knockdown attenuated disease progression [51]	Preclinical disease-mechanism relevance	Human evidence limited to expression data
Raptin-associated obesity and night-eating syndrome	Human cohort associations with sleep-deficiency-associated obesity and night-eating syndrome [21]	Raptin suppressed feeding and gastric emptying in vivo [21]	Candidate endocrine translational relevance	No independent Raptin-specific validation

Note: The translational labels are descriptive and non-hierarchical and reflect the type and maturity of the available evidence. “Candidate” denotes a potentially clinically relevant human association lacking independent or prospective validation; “preclinical” denotes RCN2-directed functional evidence for which the specific translational implication remains clinically unvalidated; “mechanistic” denotes experimentally supported disease-relevant function without demonstrated clinical utility; “human-associated and experimentally supported” denotes concordant human observational and RCN2-directed functional evidence that remains clinically unvalidated; and “context-dependent” denotes evidence that varies across sample types or experimental settings. Current translational interpretations and principal limitations represent the author’s evidence-calibrated appraisal based on the cited evidence and should not be interpreted as a validated clinical grading system. Abbreviations: CAD, coronary artery disease; CRC, colorectal cancer; ESCC, esophageal squamous cell carcinoma; HCC, hepatocellular carcinoma; NPC, nasopharyngeal carcinoma; OSCC, oral squamous cell carcinoma; PBMC, peripheral blood mononuclear cell; PDAC, pancreatic ductal adenocarcinoma; RCN2, reticulocalbin-2.

## Data Availability

The datasets supporting this review are included within the article and its Supplementary Materials.

## Supplementary Materials

The following supporting information can be downloaded at: https://www.mdpi.com/article/10.3390/genes17101226/s1, Table S1: Database-specific search strategies and screening counts for the RCN2 literature review. File S1: Record-level BioGRID raw export for RCN2. The *RCN2* gene-specific dataset was downloaded from BioGRID release 5.0.260 on 19 August 2026. The BioGRID Raw Export worksheet retains the original BioGRID interaction records and source columns without filtering, deduplication, reclassification, or alteration of record-level values. File S2: Partner-level summary and review-specific prioritization of BioGRID-derived candidate associations with RCN2. The summary was generated from the *RCN2* gene-specific export from BioGRID release 5.0.260, accessed on 19 August 2026. Tier A comprised human candidate partners supported by at least two distinct publication identifiers or by at least one interaction record designated by BioGRID as low throughput. Tier B comprised candidates that did not meet the Tier A criteria but were reported using at least two distinct experimental systems. Tier C comprised all remaining human candidate partners. These categories were used solely to organize and prioritize database-derived associations and should not be interpreted as a validated evidence-grading system or as evidence of direct binding, biological causality, or physiological relevance.
