# Supplementary material for: Reticulocalbin-2 (RCN2) as a Context-Dependent CREC-Family Modulator: Gene Expression and Regulation, Molecular Biology, Disease Relevance, and Translational Challenges
Source: Genes (Basel). 2026 Oct 2;17(10):1226. doi: 10.3390/genes17101226 (PMC13704534; doi:10.3390/genes17101226)
Supplement: Supplementary file 1 [file genes-17-01226-s001.zip › Table S1.pdf]

Supplementary Table S1. Database-specific search strategies and screening counts for the RCN2 literature review

Database	Query ID	Exact search string	Date run	Records retrieved	Records retained	Notes
PubMed	PM-Core-1	"RCN2" OR "reticulocalbin-2" OR "reticulocalbin 2" OR "ERC-55" OR "E6-binding protein" OR "E6BP" OR "VAF1" OR "TCBP49" OR "taipoxin-associated calcium-binding protein 49" OR "Raptin"	23 September 2026	119	78	Core search; no filters applied. Retained: 63 RCN2-specific and 15 contextual/background records
PubMed	PM-Targeted-1	("RCN2" OR "reticulocalbin-2" OR "reticulocalbin 2" OR "ERC-55" OR "E6-binding protein" OR "E6BP" OR "VAF1" OR "TCBP49" OR "taipoxin-associated calcium-binding protein 49" OR "Raptin") AND ("protein interaction" OR "protein interactions" OR calcium OR "endoplasmic reticulum" OR stress OR cancer OR tumor OR vascular OR atherosclerosis OR calcification OR inflammation OR bone OR cartilage OR metabolism OR neurological OR neurocognitive OR biomarker OR prognostic OR therapeutic)	23 September 2026	106	N/A	Targeted concept search; no filters applied
Web of Science Core Collection	WOS-Core-1	"RCN2" OR "reticulocalbin-2" OR "reticulocalbin 2" OR "ERC-55" OR "E6-binding protein" OR "E6BP" OR "VAF1" OR "TCBP49" OR "taipoxin-associated calcium-binding protein 49" OR "Raptin"	23 September 2026	126	82	Core Topic search; no filters applied. Retained: 67 RCN2-specific and 15 contextual/background records
Web of Science Core Collection	WOS-Targeted-1	("RCN2" OR "reticulocalbin-2" OR "reticulocalbin 2" OR "ERC-55" OR "E6-binding protein" OR "E6BP" OR "VAF1" OR "TCBP49" OR "taipoxin-associated calcium-binding protein 49" OR "Raptin") AND ("protein interaction" OR "protein interactions" OR calcium OR "endoplasmic reticulum" OR stress OR cancer OR tumor OR vascular OR atherosclerosis OR calcification OR inflammation OR bone OR cartilage OR metabolism OR neurological OR neurocognitive OR biomarker OR prognostic OR therapeutic)	23 September 2026	95	N/A	Targeted Topic search; no filters applied

Database	Query ID	Exact search string	Date run	Records retrieved	Records retained	Notes
Google Scholar	GS-Core-1	"RCN2" OR "reticulocalbin-2" OR "reticulocalbin 2" OR "ERC-55" OR "E6-binding protein" OR "E6BP" OR "VAF1" OR "TCBP49" OR "taipoxin-associated calcium-binding protein 49" OR "Raptin"	23 September 2026	~5,200	9	Core search; no filters applied; result count approximate and dynamic. Retained: 9 additional unique records, comprising 3 RCN2-specific and 6 contextual/background records

Note: PubMed and Web of Science retained counts overlap and should not be summed. Targeted searches were subsets of the corresponding core searches and served as sensitivity checks; they were not screened separately or added to the screening-flow counts. Google Scholar was used as a supplementary broad-discovery source; the first 200 relevance-ranked results were screened, and its displayed result count (~5,200) was approximate and dynamic. The Google Scholar retained count represents additional unique records identified after cross-database checking; Extended targeted Boolean searches in Google Scholar yielded predominantly low-specificity results and were therefore not used for formal screening counts.
